# COSA-1 mediated pro-crossover complex formation promotes meiotic crossing over in *C. elegans*

**DOI:** 10.1093/nar/gkae130

**Published:** 2024-02-27

**Authors:** Yuejun Yang, Nan Wang, Guoteng Liu, Wencong Nan, Bin Wang, Anton Gartner, Hongtao Zhang, Ye Hong

**Affiliations:** Shandong Provincial Key Laboratory of Animal Cell and Developmental Biology, School of Life Sciences, Shandong University, Qingdao, Shandong 266237, China; Shandong Provincial Key Laboratory of Animal Cell and Developmental Biology, School of Life Sciences, Shandong University, Qingdao, Shandong 266237, China; Shandong Provincial Key Laboratory of Animal Cell and Developmental Biology, School of Life Sciences, Shandong University, Qingdao, Shandong 266237, China; Shandong Provincial Key Laboratory of Animal Cell and Developmental Biology, School of Life Sciences, Shandong University, Qingdao, Shandong 266237, China; National Key Laboratory of Non-food Biomass and Enzyme Technology, Guangxi Academy of Sciences, Nanning, China; Institute for Basic Sciences Center for Genomic Integrity, Graduate School for Health Sciences and Technology and Department for Biological Sciences, Ulsan National Institute of Science and Technology, Ulsan, Republic of Korea; Shandong Provincial Key Laboratory of Animal Cell and Developmental Biology, School of Life Sciences, Shandong University, Qingdao, Shandong 266237, China; Shandong Provincial Key Laboratory of Animal Cell and Developmental Biology, School of Life Sciences, Shandong University, Qingdao, Shandong 266237, China

## Abstract

Accurate chromosome segregation during meiosis requires the establishment of at least one crossover (CO) between each pair of homologous chromosomes. CO formation depends on a group of conserved pro-CO proteins, which colocalize at CO-designated sites during late meiotic prophase I. However, it remains unclear whether these pro-CO proteins form a functional complex and how they promote meiotic CO formation *in vivo*. Here, we show that COSA-1, a key component required for CO formation, interacts with other pro-CO factors, MSH-5 and ZHP-3, via its N-terminal disordered region. Point mutations that impair these interactions do not affect CO designation, but they strongly hinder the accumulation of COSA-1 at CO-designated sites and result in defective CO formation. These defects can be partially bypassed by artificially tethering an interaction-compromised COSA-1 derivate to ZHP-3. Furthermore, we revealed that the accumulation of COSA-1 into distinct foci is required to assemble functional ‘recombination nodules’. These prevent early CO-designated recombination intermediates from being dismantled by the RTEL-1 helicase and protect late recombination intermediates, such as Holliday junctions, until they are resolved by CO-specific resolvases. Altogether, our findings provide insight into COSA-1 mediated pro-CO complex assembly and its contribution to CO formation.

## Graphical Abstract

**Graphical Abstract:**
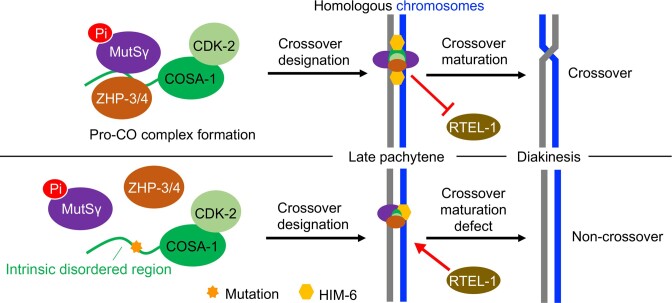

## Introduction

Proper segregation of chromosomes during the first meiotic division requires crossover (CO) formation between maternal and paternal homologous chromosomes. CO formation begins with programmed DNA double-strand breaks (DSBs) generated by the topoisomerase-like Spo11 protein (1). DSBs are then resected to produce 3′ single-stranded DNA overhangs that will invade a homologous template, forming D-loop intermediates. Most of these intermediates are disassembled by helicases, such as Rtel1 or Sgs1/BLM, and repaired without CO formation, likely via the synthesis-dependent strand annealing pathway (SDSA) (2). Other intermediates are resolved via intersister-chromatid recombination (3,4). Only a small proportion of intermediates are stabilized by pro-CO factors, which lead to the formation of Holliday junction (HJ) intermediates that link maternal and paternal chromosomes and can be resolved as CO by structure-selective endonucleases (5,6).

The frequency and position of COs are highly regulated (7,8). Excess or insufficient COs impede accurate chromosome segregation during meiosis (9,10). COs are not randomly distributed along chromosomes (11). COs near the middle of chromosome arms are more capable of promoting proper chromosome segregation than COs near telomeres (12). In addition, a CO in one location prevents the occurrence of another CO nearby, a phenomenon known as CO interference (13,14). In many eukaryotes, CO can be divided into two classes. Class I COs are sensitive to interference and account for most CO formed in plants and mammals, while class II COs do not exhibit interference and require the Mus81-Eme1 endonuclease (15,16).

Pro-CO proteins play critical roles in class I CO formation (17). Genetic screens initially identified pro-CO factors for genes that, when mutated, lead to a severe decrease in CO frequency (18,19). These pro-CO factors include several proteins, such as the Msh4/Msh5 complex (MutSγ), Zip3 family members (Zip3, ZHP-3, Hei10, Rnf212), and the cyclin-related protein CNTD1 (the mammalian ortholog of *Caenorhabditis elegans* COSA-1) (20–22). The Msh4/Msh5 heterodimer recognizes HJs and forms a meiosis-specific sliding clamp, embracing two homologous chromosomes to facilitate CO formation (23,24). Zip3 family members have an N-terminal RING finger domain commonly associated with ubiquitin or SUMO ligase activity (25). COSA-1/CNTD1 is conserved specifically in Metazoa and has been shown to congregate into CO designation sites and to be required for CO maturation by forming a complex with CDK-2 (26–28). Loss of CNTD1 and CDK2 in mice results in a dramatically decreased number of COs and infertility (27,29).

In *C. elegans*, typically, only one CO occurs for each pair of homologous chromosomes (30). *C. elegans* pro-CO factors show dynamic localization during meiotic prophase (31). For example, ZHP-3 and its paralog ZHP-4 initially appear as puncta on the synaptonemal complex (SC) in early pachytene, then spread along the entire length of the SC and finally become restricted to the six CO-designated sites in late pachytene (32–34). Similarly, the localization of other pro-CO factors is first widespread in each meiotic nucleus before finally becoming confined to designated CO sites. These pro-CO factors colocalize at CO-designated sites, and their localization is interdependent. Interestingly, careful examination of the organization of proteins assembled at the CO-designated sites by high-resolution cytological analysis revealed a distinct architecture, with COSA-1 being detected at the center of a cruciform structure defined by the orthogonally oriented MSH-5 and HIM-6 doublets (31). Once a recombination intermediate has been marked and stabilized by pro-CO factors, it may undergo resolution into COs involving two redundant pathways composed of (a) the structure-selective endonucleases MUS-81 and SLX-1, as well as (b) the XPF-1 nuclease and HIM-6 the nematode BLM helicase (35–37). Both resolvase activities need the SLX-4 scaffold protein (38,39). Without both pathways, most chromosomes fail to form bivalent structures. Instead, chromatin bridges loosely connect homologs due to unresolved recombination intermediates (35).

The functional interdependence and colocalization of pro-CO factors indicate that they may form a complex at the CO-designated sites. However, the physical interactions of CO factors and the significance thereof have not been investigated. Here, we describe the interactions between COSA-1 and MSH-5 as well as ZHP-3 via the COSA-1 N-terminal disordered region. These interactions are essential for the accumulation of COSA-1 at the CO-designated sites and, consequently, for CO formation. Our data also provide *in vivo* evidence supporting the role of the pro-CO complex in stabilizing CO-designated recombination intermediates at both early and late stages of meiotic recombination.

## Materials and methods

### *C. elegans* strains and maintenance

All strains in this study were maintained on NGM (nematode growth medium) plates seeded with OP50 bacteria at 20 °C. All *C. elegans* strains were derived from a Bristol N2 background. The *OLLAS::cosa-1*, *msh-5-T1009A::AID::3×HA* and *cdk-2::AID::3×FLAG* strains were generated respectively using the same tagging strategy as previously reported (40,41). To generate strains expressing COSA-1::3×HA::TurboID, MSH-5::AID::3×HA, OLLAS::COSA-1::3×FLAG, variants of OLLAS::COSA-1::3×FLAG, OLLAS::COSA-1-4A::3×FLAG::GFP-nanobody, 3×FLAG::DSB-2, ZHP-3::AID::3×HA and RTEL-1::AID::3×HA, worms were injected with CRISPR/Cas9 injection mixture (10 μl volume), including 0.2 μl Cas9 (IDT, 10 μg/μl stock) complexed with 1 μg sgRNA, 10 ng/μl pCFJ90 and 10 ng/μl pCFJ104 injection marker plasmids, and a melting dsDNA donor cocktail (30 ng/μl) or single-stranded templates (30 ng/μl) in the final injection mixture as a repair template (Supplementary Table S1) (42,43). Progenies were genotyped by PCR to detect insertion edits and validated by sequencing. The strains were outcrossed with N2 wild type at least 3 times prior to analysis. Supplementary Table S2 summarizes all mutations and strains used in this study.

### TurboID-based enzymatic protein labeling and extraction of biotinylated proteins from *C. elegans*

At least 500 μl synchronous adult worms (N2, *GFP::him-6*, *cosa-1::3×HA::TurboID; GFP::him-6*,*cdk-2::AID::3×FLAG*, *cosa-1::3×HA::TurboID* and*cdk-2::AID::3×FLAG; cosa-1::3×HA::TurboID*) were collected and washed with M9 buffer. Two volumes of RIPA buffer (1% Triton X-100, 1 mM EDTA, 0.5% sodium deoxycholate, 0.1% SDS, 150 mM NaCl, 50 mM Tris–HCl pH7.4) supplemented with 1 mM PMSF were added to resuspend the worms and frozen worm ‘popcorn’ was then prepared by liquid nitrogen as previously reported (44). After the samples were ground and completely thawed, SDS and DTT were added to a final concentration of 1% and 10 mM, respectively. The samples were immediately incubated at 95°C for 10 min, treated by sonication (15% continuous output, SCIENTZ, JY92-IIN), and then adjusted to 2 M urea using a stock solution (8 M urea, 1% SDS, 50 mM Tris–HCl pH7.4, 150 mM NaCl) and centrifuged at 13000 rpm for 20 min. The clear supernatant between the pellet and the surface lipid layer was transferred to a new tube and then loaded twice onto Zeba spin desalting columns (7K MWCO) (Thermo Fisher) to remove free biotin. Dynabeads MyOne streptavidin T1 (Invitrogen, 80 μl/1 ml worms) were finally added to samples to extract biotinylated proteins by incubation at room temperature overnight. Beads were then washed three times with wash buffer (150 mM NaCl, 1 mM EDTA, 2% SDS, 50 mM Tris–HCl, pH7.4). Beads were boiled in sample buffer with 80 mM biotin (Sangon) for 15 min.

### Yeast two-hybrid analysis

The yeast two-hybrid assay was performed according to (45). Full-length coding sequences for pro-CO factors (COSA-1, ZHP-3, MSH-5, MSH-4, HIM-6, and CDK-2) and various COSA-1 mutants were cloned into plasmid pGADT7 or pGBKT7, transformed into yeast strain AH109. Positive colonies were selected on a medium lacking tryptophan (-Trp) and leucine (-Leu). Positive colonies were resuspended in 1 ml 1xPBS and serial dilutions were plated on -His -Trp -Leu and -Trp -Leu solid media at 25°C for 3 or 4 days.

### Protein depletion by combination of Auxin-inducible degradation and RNAi

Auxin-inducible degradation of RTEL-1 from the *C. elegans* germline was performed as previously described (46,47). Briefly, auxin plates were prepared by diluting 400 mM K-NAA, a synthetic auxin analog into NGM to a final concentration of 3 mM. To achieve the strongest possible depletion of target protein, we combined AID depletion with RNAi. NGM agar was cooled to 55°C and supplemented with K-NAA, IPTG, tetracycline, and ampicillin just before pouring plates to a final concentration of 3 mM, 1 mM, 25 μg/ml and 25 μg/ml, respectively. RNAi plasmids for *rtel-1* knockdown were constructed by inserting a 1027 bp coding sequence of *rtel-1* into the L4440 vector. *Escherichia coli* strain HT115 was used for RNAi. Cultures containing the RNAi plasmid or the corresponding empty vector were cultured overnight at 37°C in the presence of 25 μg/ml tetracycline hydrochloride and ampicillin and then cultured at 20°C and 160 rpm for 7 h in the presence of 1 mM IPTG to induce dsRNA synthesis. 400 μl culture supplemented with 3 mM K-NAA was added to the plates. L4 larvae were fed on K-NAA and IPTG plates at 20°C for 52 h prior to cytological analysis.

### Cytological procedures

Young adult hermaphrodites (24 h post L4) were dissected in 7 μl dissection buffer (25 mM HEPES pH 7.4, 118 mM NaCl, 48 mM KCl, 2 mM EDTA, 5 mM EGTA, 0.1% Tween-20) and briefly fixed in 1% formaldehyde. Gonads were flash-frozen in liquid nitrogen, freeze-cracked, and put in -20°C methanol for 10 min. Wash slides three times in 1× PBST (0.1% Tween 20) and then blocked with 1% BSA for 30 min at room temperature. Samples were incubated with primary antibodies overnight at 4°C at the indicated dilutions: mouse anti-FLAG (1:600, Sigma F1804), mouse anti-HA (1:600, BioLegend 16B12), GFP booster (1:400, Chromotek #gb2AF488), guinea pig anti-HTP-1 (1:300, generated by ABclonal Technology), rabbit anti-HIM-3 (1:300, generated by ABclonal Technology), rabbit anti-SYP-1 (1:300, generated by ABclonal Technology), rabbit anti-RAD-51 (1:1000, generated by ABclonal Technology), rabbit anti-OLLAS (1:500, Genscript A01658), and rabbit anti-MSH-5 pT1009 (1:300, obtained from Yumi Kim). Slides were washed with PBST and incubated with secondary antibodies at a 1:1000 dilution (Invitrogen Alexa 488, Alexa 594 or Alexa Fluor Plus 647) and DAPI (1:20) (Sangon Biotech E607303) for 2 h at room temperature. Slides were rewashed in PBST and mounted in an antifade mounting medium (Vectashield H-1000). Slides were imaged using LSM 900 with Airyscan (ZEISS) equipped with a 63× oil immersion and 1.4 NA objective or Olympus SpinSR10 with a 60× oil immersion and 1.42 NA objective. DAPI bodies in the nuclei of −1 to −3 diakinesis oocytes were counted.

Chromosome spreading of *C. elegans* germ cell nuclei was performed as previously described (31,48).The gonads of 20 adult worms were dissected in 5 μl high (85% v/v Hank's Balanced Salt Solution (HBSS, Life Technology, 24020-117) with 0.1% v/v Tween-20) (Figure 5D) or low solution (0.2× PBS with 0.1% v/v Tween-20) (Figures 4A, 5A, C, Supplementary Figures S7A, S7B, S8A and S8B) on a 18 × 18 mm coverslip (ZEISS, thickness 0.170 ± 0.005 mm). 50 μl of spreading solution was added and gonads were immediately distributed over the whole coverslip. Coverslips were put in 6 cm plates and left to dry overnight at room temperature, washed for 20 min in methanol at -20°C and rehydrated by washing 3 times for 10 min in 1× PBST. After rehydrating, samples were processed for immunofluorescence using antibodies at the concentrations listed above. Ultra-high resolution imaging with 250 nm spaced Z-stacks was performed using ZEISS Elyra 7 microscope SIM equipped with a 63× 1.40 NA objective. Spreading solution (for one coverslip, 50 μl): 32 μl of fixative (4% w/v paraformaldehyde and 3.2 ± 3.6% w/v sucrose in water), 16 μl of Lipsol solution (1% v/v Lipsol in water) and 2 μl of sarcosyl solution (1% w/v of sarcosyl in water, Sigma-Aldrich L7414).

### Meiotic crossover recombination frequency assay

Meiotic CO recombination frequencies were determined as described, using six insertion–deletion polymorphisms on Chromosome Ⅱ that differ between N2 Bristol and CB4856 Hawaii (49). Strains used to determine CO recombination assays were crossed into Hawaii to obtain mutant strains carrying ChrⅡ homozygous for Hawaii DNA. Homozygous mutant males with ChrⅡ homozygous Hawaii were then crossed with hermaphrodites of identical genotype in the N2 Bristol background to obtain mutant strains heterozygous for Hawaii. F1 cross-progeny hermaphrodites were then crossed with males of CB5584, a *myo-2::GFP* expressing strain, allowed to lay eggs for 24–48 h. The F2 offspring, which express high levels of green fluorescent protein in pharyngeal muscles, were picked for genotyping and analyzed for CO recombination by PCR. Primers used: Chromosome II: genetic position (gp) -15.4 B5:primer 1:5′-AACGACGCGATGCTATGGAT-3′, primer 2:5′-TGGAATTGAAACAGAACTCAGC-3′, N2:1100 bp, CB4856:824 bp; gp -6.3 B7: primer 1:5′-ATTTGGGTGGGAACTGGAGG-3′, primer 2:5′-GCGTGCAGACATAAGATAGGG-3′, N2:634 bp, CB4856:425 bp; gp 0.1 B10: primer 1:5′-ACCAGCAATAGGTCAAGGTCT-3′, primer 2:5′-CACGTCATTCGCCAGTCAAA-3′, N2:819 bp, CB4856:484 bp; gp 5.7 C3:primer 1:5′-ACATGGGAGCGACGGTTTTA-3′, primer 2:5′-CCCGACACCATAACACAACA-3′, N2:933 bp, CB4856:448 bp; gp 17.5 C5:primer 1:5′-AGCCGTTACTCGCCATGAAA-3′, primer 2:5′-GCCAAACATCGGTCATCGGA-3′, N2:959 bp, CB4856:744 bp; gp 23.1 C6:primer 1:5′-TTGTGTGCAAACACCGTCAC-3′, primer 2:5′-TCGGTCCGAAGGCAATCAAA-3′, N2:1220 bp, CB4856:811 bp.

### Analysis of progeny viability and incidence of males

L4 hermaphrodites were singled on NGM plates. The worms were transferred onto fresh plates every 12 h for three or four consecutive days until no offspring were produced. The number of eggs was counted after each transfer. Unhatched eggs were counted 24 h later, and the percentage of progeny viability was calculated as the total number of viable progenies divided by the total number of eggs. The brood size was determined as the total number of progenies divided by the number of adult worms singled on the plates. The incidence of males was calculated as the number of males divided by the total number of viable progenies.

### Immunoblots

Protein samples were separated by SDS-PAGE and transferred onto PVDF membranes (Millipore IPVH00010). Membranes were blocked in 5% milk in PBST for 1 h at room temperature and incubated with primary antibodies overnight at 4°C with agitation. Primary antibodies were used in the following dilutions: mouse anti-HA (1:2000, Cell Signaling, 6E2), mouse anti-HA (1:2000, BioLegend 16B12), rabbit anti-GFP (1:2000, ABclonal Technology AE011), mouse anti-FLAG (1:2000, Sigma F1804), mouse anti-actin (1:3000, Proteintech, #66009-1-Ig), rabbit anti-HIM-3 (1:1000, generated by ABclonal Technology) and rabbit anti-MSH-5 (1:1000, generated by ABclonal Technology).

### Statistics

Distributions of DAPI-stained bodies were statistically tested by the Kruskal–Wallis test. The FLAG (COSA-1) signal intensity in nuclei was tried by the Mann–Whitney *U* test, while progeny viability and incidence of males by *t*-test. *P*< 0.05 was considered significant.

## Results

### COSA-1 interacts with ZHP-3 and MSH-5

**Figure 1.**
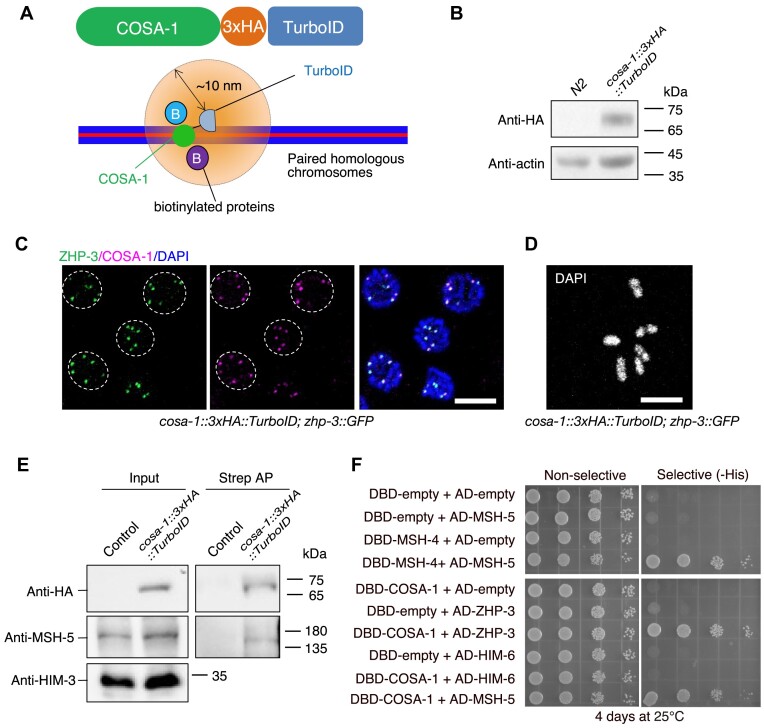
COSA-1 interacts with MSH-5 and ZHP-3, but not with HIM-6. (**A**) Illustration of TurboID-mediated proximity labeling in *cosa-1::3×HA::TurboID* knock-in *C. elegans* strain. (**B**) Western blot analysis of expression of COSA-1::3×HA::TurboID fusion protein *in vivo*. (**C**) Colocalization of COSA-1::3xHA::TurboID with ZHP-3 at the CO-designated sites. Scalebar, 5 μm. (**D**) DAPI- stained oocyte from *cosa-1::3×HA::TurboID* knock-in strain. Scalebar, 5 μm. (**E**) Biotinylated MSH-5 proteins are detected by western blotting with antibodies against MSH-5. Proteins biotinylated by TurboID were enriched with streptavidin beads by affinity purification (AP) and examined by western blotting. (**F**) Analysis of interactions among pro-CO proteins by the yeast two-hybrid system.

We wanted to understand if the colocalization of pro-CO proteins at the CO-designated sites is mediated by protein-protein interactions and, if so, to test the functional relevance of these *in vivo*. Attempting to test the interaction between COSA-1 and MSH-5 by co-immunoprecipitation (co-IP), we noticed that MSH-5 could be barely solubilized by the lysis buffer commonly used for co-IP experiments (Supplementary Figure S1). We thus turned to proximity labeling and yeast two-hybrid assays as alternative strategies. BioID and derivative procedures TurboID and miniTurbo take advantage of a biotin ligase being fused to a bait-protein, facilitating the biotinylation of proteins residing in close proximity (50). This methodology has been proven effective in detecting weak or transient protein-protein interactions (51). Additionally, it is particularly useful for insoluble proteins as biotin-labeled proteins can be easily purified even under harsh denaturing conditions. We engineered a *3×HA::TurboID* just before the stop codon of the endogenous *cosa-1* locus using CRISPR/Cas9-mediated genome editing (Figure 1A, B). In contrast with *cosa-1* null alleles, which exhibit 97% lethality (26), the resulting knock-in strain was indistinguishable from wild type for progeny viability. However, it showed a decreased brood size and a weak Him (high incidence of males) phenotype, suggesting that the fusion may affect the localization, function, or protein-protein interaction of COSA-1 (Supplementary Figure S2A-S2C). To ensure that the COSA-1 fused to TurboID remains physiologically functional and that its nature still reflects the endogenous COSA-1, we checked the localization of COSA-1::3×HA::TurboID and CO formation in the knock-in strain. Cytological analyses showed that COSA-1::3×HA::TurboID colocalized with ZHP-3 at CO-designated sites and that six bivalents formed in the diakinesis oocytes, indicating that this fusion protein can be recruited to the CO-designated sites and that it is largely functional for CO formation (Figure 1C, D and Supplementary Figure S2D). We next carried out streptavidin affinity pull-downs to enrich TurboID-biotinylated proteins. CDK-2, a known binding partner of COSA-1, served as a positive control (Supplementary Figure S2E) (40). MSH-5 and HIM-6 were identified among the streptavidin-purified proteins (Figure 1E, Supplementary Figure S2F). Our TurboID results are consistent with previous cytological studies, showing colocalization between COSA-1 and MSH-5 or HIM-6 (31).

We used the yeast two-hybrid system to further explore potential interactions among pro-CO proteins. As a positive control, we included MSH-4-MSH-5 and HIM-6-RMH-1, which are known to function together as heterodimers (23,52). Our results revealed that COSA-1 interacts with CDK-2 (Supplementary Figure S3A), consistent with a previous study (40). In addition, we observed interactions between COSA-1 and MSH-5, as well as ZHP-3 (Figure 1F). Although HIM-6 could be identified among the biotinylated proteins modified by COSA-1::3×HA::TurboID, we did not detect an interaction between COSA-1 and HIM-6 (Supplementary Figure S3B). We also failed to observe interactions between HIM-6 and other factors localized at CO-designated sites, such as ZHP-3/4 and MSH-4/5 (Supplementary Figure S3B). However, a negative result in a yeast two-hybrid system does not mean that the tested proteins do not interact under all conditions. On the other hand, we cannot exclude the possibility that other unidentified pro-CO proteins mediate the interaction between HIM-6 and COSA-1 or other pro-CO factors.

### The N-terminus of COSA-1 mediates the interaction with MSH-5 and ZHP-3

**Figure 2.**
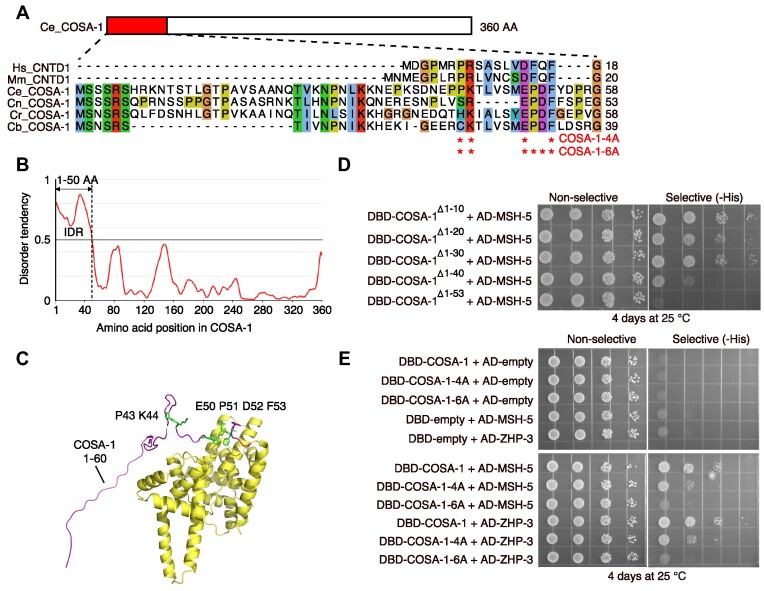
The N-terminus of COSA-1 is required for interaction with MSH-5 and ZHP-3. (**A**) Schematic of the N-terminal sequences from COSA-1 and its orthologs from different organisms. The sites of mutations are indicated. Sequences are shown as follows: Hs, *Homo sapiens* (NP_755749.2, NCBI Sequence ID); Mm, *Mus musculus* (NP_080838.1, NCBI Sequence ID); Ce, *Caenorhabditis elegans* (NP_497607.3, NCBI Sequence ID); Cn, *Caenorhabditis nigoni* (PIC38620.1, GenBank); Cr, *Caenorhabditis remanei* (XP_053587050.1, NCBI Sequence ID); Cb, *Caenorhabditis brenneri* (EGT33696.1, GenBank). (**B**) The intrinsically disordered region (IDR) of COSA-1 was analyzed by IUPRED3 and the propensity for disorder was drawn as a solid line. Values above 0.5 indicate an IDR. (**C**) The structure of COSA-1 predicted by Alphafold2 revealed a long flexible N-terminus. The sites of mutations are indicated. (**D**) The yeast two-hybrid system was used to further refine the region of COSA-1 required for the interaction with MSH-5. (**E**) Analysis of interaction between COSA-1-4A or 6A and ZHP-3 or MSH-5 by yeast two-hybrid system.

We next sought to investigate how these proteins interact with each other. CNTD1, the mammalian ortholog of COSA-1, is predicted to have two isoforms (27,53). The predominant form, which lacks the N-terminal 85 amino acids, failed to interact with crucial factors for CO formation, such as MSH4, MSH5, and CDK2, hinting that the N-terminus may be necessary for the interaction between CNTD1 and other pro-CO factors (53). Sequence analysis and structure prediction revealed that COSA-1 has a long N-terminus containing an intrinsically disordered region (IDR) spanning residue 1–50 (Figure 2A-C). IDRs are often found to mediate protein/protein interactions (54). To refine the region of COSA-1 required for interactions with MSH-5 and ZHP-3, we tested the interaction between MSH-5 and a series of COSA-1 N-terminal truncations: COSA-1^Δ1–10^, COSA-1^Δ1–20^, COSA-1^Δ1–30^, COSA-1^Δ1-40^ and COSA-1^Δ1–53^ using yeast two-hybrid assays. While the truncated COSA-1^Δ1–10^, COSA-1^Δ1–20,^ and COSA-1^Δ1–30^ could interact with MSH-5 and ZHP-3, COSA-1^Δ1-40^ showed dramatically decreased interactions with these two proteins (Figure 2D). The interaction got further weakened to an undetectable level upon deleting 13 additional amino acids from the N-terminus (Figure 2D), suggesting that the N-terminal 53 amino acids of COSA-1 play a vital role in the interactions with MSH-5 and ZHP-3.

To further narrow down the amino acids involved in the interaction of COSA-1 with MSH-5 or ZHP-3, we aligned COSA-1 and its orthologs and identified four conserved residues (P43, K44, E50 and F53) in the N-terminus (Figure 2A). In addition, two amino acids, P51 and D52, are specifically conserved in worms (Figure 2A). We introduced two sets of mutations in the conserved residues of COSA-1 and examined the effects on its interaction with pro-CO proteins. COSA-1-4A, carrying four residues (P43, K44, E50 and F53) changed to alanine, showed weaker interaction with MSH-5 and ZHP-3 than wild-type COSA-1, while mutation of all six residues (COSA-1-6A) nearly abolished the interactions (Figure 2E). However, both COSA-1-4A and COSA-1-6A retained the interactions with their kinase partner CDK-2 (Supplementary Figure S3A), suggesting that the N-terminus of COSA-1 is not involved in the interaction with CDK-2.

### The N-terminus of COSA-1 is required for the accumulation of COSA-1/CDK-2 at CO-designated sites and CO formation

**Figure 3.**
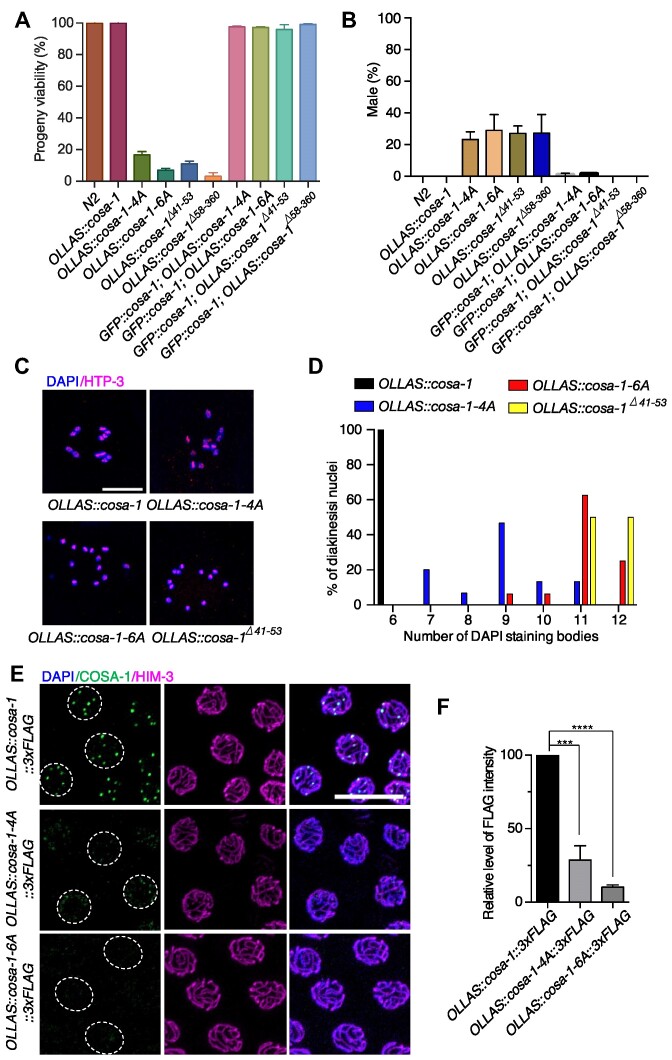
*cosa-1-4A and cosa-1-6A* mutants are compromised for CO formation. (**A**) Quantification of progeny viability and (**B**) frequencies of male offspring among the progeny of the indicated genotypes. (**C**) Representative full projections of diakinesis nuclei of the indicated genotypes stained with DAPI. Scalebar, 10 μm. (**D**) Quantitative analysis of the number of DAPI-stained bodies in diakinesis nuclei for the indicated genotypes. (**E**) Representative images of late pachytene nuclei of *cosa-1-4A* and *cosa-1-6A* mutants stained with antibody against FLAG (green), HIM-3 (magenta) and DAPI (blue). While wild-type COSA-1 concentrated to ∼6 bright foci at CO-designated sites, only weak dimmer foci could be detected in *cosa-1-4A* and *cosa-1-6A* mutants. Scalebar, 10 μm. (**F**) Quantification of the FLAG signal (COSA-1) in nuclei of the indicated genotypes. Statistical analyses were conducted using *t***-**test, *** *P*< 0.005, **** *P*< 0.001.

To investigate the biological significance of the N-terminus of COSA-1, we generated worm strains expressing COSA-1-4A and COSA-1-6A by CRISPR/Cas9, appending an N-terminal OLLAS tag to all *cosa-1* derivates for visualization of the respective proteins. The *OLLAS::cosa-1* strain was fully functional (Figure 3A, B). As previously reported (41), *OLLAS::cosa-1* foci first appear in early pachytene, then rise in abundance during mid pachytene, and finally reach a plateau at 6 foci per nucleus in late pachytene (Supplementary Figure S4A–C). Similar to a newly created *cosa-1^Δ58–360^* mutant in this study, which removes the entire cyclin domain and is predicted to be a null allele, the *cosa-1-4A* and *cosa-1-6A* mutations lead to a dramatically decreased progeny viability (16.6% and 6.7%, respectively) and a Him (high incidence of males) phenotype (23.2% and 29%, respectively) among surviving progenies (Figure 3A, B). Both phenotypes could be rescued by transgenic expression of a wild-type COSA-1 (Figure 3A, B). Western blotting analysis showed that COSA-1-4A was expressed at the expected size, while the band of COSA-1-6A was slightly lower than COSA-1-4A and wild-type COSA-1 (Supplementary Figure S4E). The expression level of both proteins was modestly reduced (73.1% and 80.8% of the wild-type level, respectively) (Supplementary Figure S4D).

The Him phenotype prompted us to check if mutation of these interaction sites at the N-terminus of COSA-1 results in defects in CO formation. Wild-type diakinesis oocytes contain 6 DAPI-stained bodies, corresponding to six pairs of homologs held together by chiasmata. In *cosa-1-4A* and *cosa-1-6A* mutants, 7–12 DAPI-stained bodies were observed, with an average of 8.93 (*N* = 15, *P*< 0.0001) and 11.06 (*N*= 16, *P*< 0.05) respectively, compared with 11.6 for *cosa-1^Δ58–360^* null mutants (*N* = 25), reflecting a partial impairment of CO formation (Figure 3C, D). Similarly, *cosa-1^Δ40–53^* mutants, which had an in-frame deletion covering all 6 conserved residues, exhibited an average of 11.5 DAPI-stained bodies (*N* = 20), indicating that the N-terminus of COSA-1 is indeed required for CO formation (Figure 3C, D).

Failure in CO formation in *cosa-1-4A* and *cosa-1-6A* mutants was not caused by defects in recombination initiation, as numerous RAD-51 foci were detected in early pachytene (Supplementary Figure S5A). Most RAD-51 foci eventually disappeared at late pachytene, indicating that most DSBs were repaired but were not converted into interhomolog COs (Supplementary Figure S5A). We then examined the localization of COSA-1 in those mutants during meiotic prophase. Wild-type COSA-1 localizes to 6 distinct foci per nucleus in late pachytene, corresponding to 6 CO-designated sites (Figure 3E). However, no bright COSA-1 foci were observed on chromosome tracks in the *cosa-1-4A* and *cosa-1-6A* mutants (Figure 3E, Supplementary Figure S6A, B). Instead, only faint signals could be detected within the nuclei in *cosa-1-4A* mutants (Figure 3E, F). Similarly, CDK-2 foci were also markedly reduced in the *cosa-1-4A* and *cosa-1-6A* mutants (Supplementary Figure S5B, C), indicating that the COSA-1 N-terminus is essential for the accumulation of COSA-1/CDK-2 at CO-designated sites.

### CO designation is normal in *cosa-1-4A* and *cosa-1-6A* mutants despite the defective accumulation of COSA-1 at the CO designation sites

**Figure 4.**
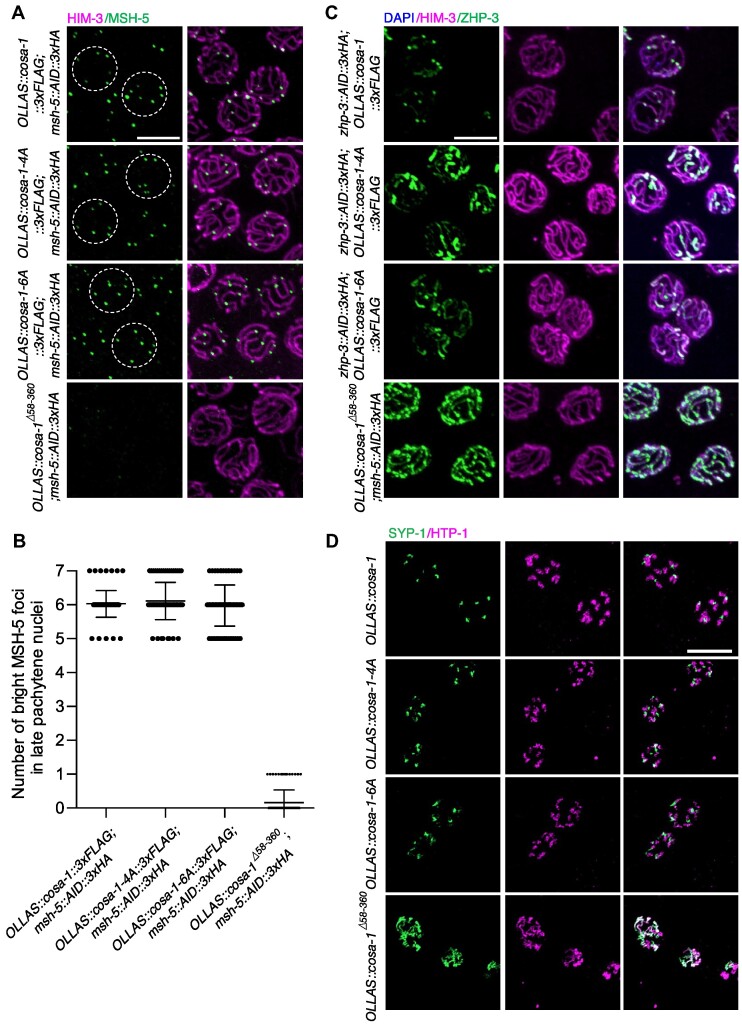
*cosa-1-4A and cosa-1-6A* mutations do not perturb CO designation. (**A**) Dissected gonads were spread and representative images of late pachytene nuclei of *cosa-1-4A* and *cosa-1-6A* mutants stained with antibodies against HA (green) and HIM-3 (magenta). Scalebar, 5 μm. (**B**) Quantitative analysis of the number of bright MSH-5 foci in late pachytene nuclei for the indicated genotypes. (**C**) Representative images of late pachytene nuclei of the indicated genotypes stained with antibodies against HA (green) and HIM-3 (magenta), counterstained with DAPI (blue). Scalebar, 5 μm. (**D**) Representative images of diplotene nuclei of the indicated genotypes stained with antibodies against SYP-1 (green) and HTP-1(magenta). Scalebar, 10 μm.

Pro-CO factors colocalize at CO-designated sites and exhibit interdependence for their localizations (26). Since *cosa-1-4A* and *cosa-1-6A* mutants displayed a defective COSA-1 accumulation at the CO-designated sites, we investigated whether the localizations of MSH-5 and ZHP-3 were affected. Normally, MSH-5 first appears in zygotene as numerous foci and is then removed from most repair sites during the early-to-late pachytene transition (31). MSH-5 concentrates at the CO-designated sites in late pachytene to promote CO formation. ZHP-3 also shows dynamic localization. ZHP-3 initially localizes along the SC and becomes gradually concentrated at the CO-designated sites in the late prophase (32). While late pachytene MSH-5 foci were diminished or undetectable in *cosa-1^Δ58–360^* mutant, MSH-5 appeared normal and displayed 6 bright foci in most of the late pachytene nuclei in both *cosa-1-4A* and *cosa-1-6A* mutants as in wild type (Figure 4A, B and Supplementary Figure S7A, B).

In addition, phosphorylation of MSH-5 was not affected in *cosa-1-4A/6A* mutants when we used MSH-5^T1009^ phospho-antibody for immunostaining (Supplementary Figure S8A, B). T^1009^ is so far the only confirmed phosphorylation site on the MSH-5 C-terminal tail by CDK-2 kinase (40). To determine whether *cosa-1-4A* showed synthetic phenotypes with *msh-5* phosphomutants, we combined *cosa-1-4A* with *msh-5^T1009A^* mutation. The *OLLAS::cosa-1-4A::3×FLAG; msh-5^T1009A^::AID::3×HA* was indistinguishable from *OLLAS::cosa-1-4A::3×FLAG* in terms of progeny viability, incidence of male and bivalent formation (Supplementary Figure S9A-D). We then analyzed the dynamic localization of ZHP-3. Unlike the *cosa-1^Δ58–360^* mutant, which exhibited persistence of ZHP-3 along the length of the chromosome, ZHP-3 eventually became concentrated at CO-designated sites, although a bit delayed in *cosa-1-4A* and *cosa-1-6A* mutants (Figure 4C, Supplementary Figure S10A, B). These results indicate that mutations in the N-terminus of COSA-1 overall do not affect the ability of MSH-5 and ZHP-3 to accumulate at the CO-designated sites.

DSB-1 and DSB-2 localize to the chromosomes during early meiotic prophase and are required for DSB formation (55,56). Absence of crossover precursors or designation has been shown to induce prolonged DSB-1 or DSB-2 association with chromosome, therefore resulted in extended DSB-1 or DSB-2 zone in the germline. In wild type, DSB-2 positive nuclei comprised about 59.8% of the length from onset of DSB-2 staining to the end of the pachytene region (Supplementary Figure S11A, B). In *cosa-1^Δ58–360^* mutants, the absence of crossover precursors triggers extension of DSB-2 zone to 78.9%, consistent with previous studies (56). However, *cosa-1-4A/6A* didn’t result in extended DSB-2 staining (58.6% and 60.7% respectively) (Supplementary Figure S11A, B), suggesting that the crossover designation is largely unaffected in the *cosa-1-4A/6A* mutants.

CO designation triggers bivalent differentiation through the asymmetric disassembly of the SC during late prophase. This chromosome structure remodeling results in the formation of six cruciform bivalents with distinct long and short arms (57). To determine if there were defects in bivalent differentiation in *cosa-1-4A* and *cosa-1-6A* mutants, we stained germlines with antibodies against SYP-1 and HTP-1. HTP-1 localizes to the long arms on bivalents, while SYP-1 associates with short arms in wild-type germline. Failure in CO designation or formation usually results in the loss of asymmetric localization (58). In line with this notion, *cosa-1^Δ58–360^* null mutants retained SYP-1 and HTP-1 along the entire length of chromosomes (Figures 4D). In contrast, SYP-1 properly localized to the short arm and HTP-1 to the long arm in *cosa-1-4A* and *cosa-1-6A* mutants, indicating that bivalent differentiation was unaffected (Figure 4D). Taken together, although CO formation in *cosa-1-4A* and *cosa-1-6A* mutants was severely impaired, CO designation seems to be achieved, as shown by the accumulation of MSH-5 and ZHP-3 at CO-designated sites and successful bivalent differentiation.

### Formation of late CO-specific intermediates is compromised in *cosa-1-4A* animals

**Figure 5.**
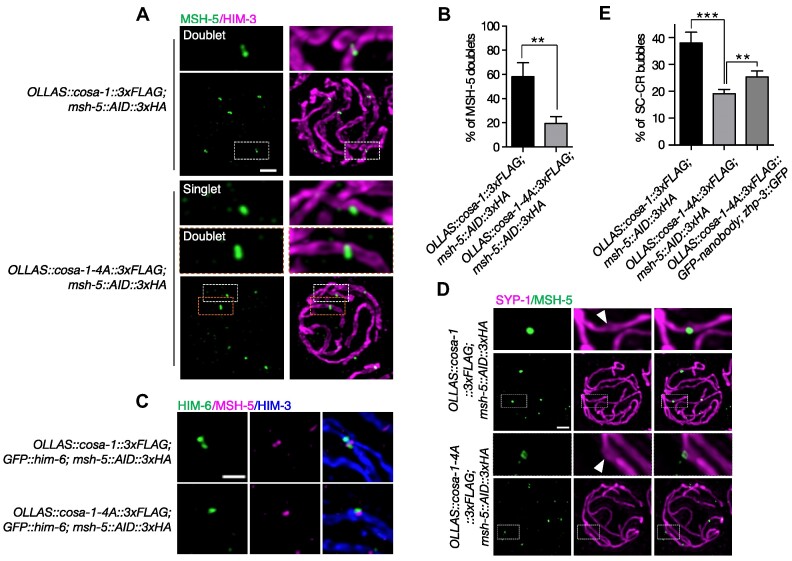
Formation of late CO-specific intermediates is compromised in *cosa-1-4A* mutant. (**A**) Representative SIM images of spread late pachytene nuclei of the indicated genotypes stained with antibodies against HA (green) to detect MSH-5 and HIM-3 (magenta). Scalebar, 1 μm. (**B**) Percentage of MSH-5 signals that are resolved as doublets at the CO-designated sites. Statistical analyses were conducted using *t***-**test, ** *P*< 0.01. (**C**) Representative SIM images of recombination sites in late pachytene. Stainings for MSH-5, HIM-6, and HIM-3 are shown. (Scale bar, 500 nm). (**D**) Representative SIM images of SC bubbles (white arrowheads) surrounding MSH-5 foci in late pachytene nuclei. Stainings for MSH-5 and SYP-1 are shown (scale bar, 1 μm). (**E**) Quantification of formation of SC bubbles at the CO-designated sites in late pachytene nuclei for the indicated genotypes. Statistical analyses were conducted using *t***-**test, ** *P*< 0.01, *** *P*< 0.005.

Recent studies have shown that MSH-5 can display different organizations, which reflect their binding to different recombination intermediates (31). Careful examination of spread nuclei using super-resolution structured illumination microscopy showed that 58% of MSH-5 signals were detected as doublets in late pachytene in wild type, compared with 19% of those in *cosa-1-4A* mutants (Figure 5A, B). The MSH-5 doublets in wild type are oriented orthogonally to the BLM doublets, indicating the presence of late CO recombination intermediates at these sites (31). However, the BLM doublets were hardly detected in *cosa-1-4A* mutants. Instead, BLM single focus adjacent to MSH-5 focus were frequently detected in *cosa-1-4A* mutants (Figure 5C). Bubble-like SC structures were reported to form at nearly half of all CO-designated sites (31). These structures are implicated to play a crucial role in facilitating CO formation by concentrating pro-CO factors. Surprisingly, MSH-5 foci in *cosa-1-4A* animals were still surrounded by bubble-like SC structures (Figure 5D). However, the number of SC bubbles at the CO-designated sites was decreased in *cosa-1-4A* mutants (Figure 5E). These data suggested that the formation of late CO-specific intermediates is compromised in *cosa-1-4A* animals.

### GFP-nanobody-mediated tethering of COSA-1-4A and ZHP-3 partially restores CO formation

**Figure 6.**
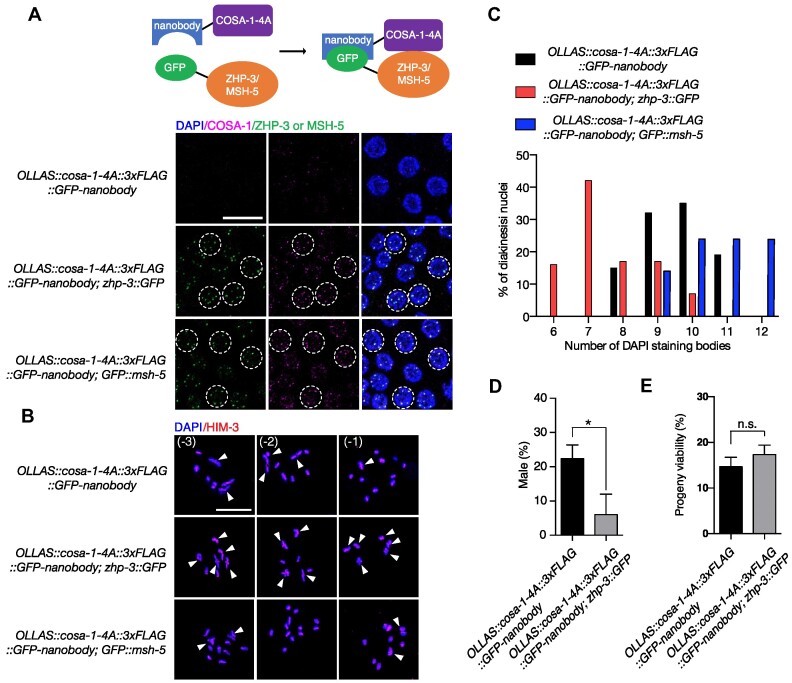
Artificially tethering interaction-compromised COSA-1 to the CO-designated sites partially restores CO formation. (**A**) Representative images of late pachytene nuclei of the indicated genotypes stained with GFP booster to detect GFP::MSH-5 or ZHP-3::GFP (green), and antibodies against FLAG (magenta) to detect COSA-1, counterstained with DAPI (blue). Scalebar, 10 μm. (**B**) Representative images of diakinesis nuclei of the indicated genotypes stained with antibodies against HIM-3 (red), counterstained with DAPI (blue). Scalebar, 10 μm. White arrowheads indicate bivalents. (**C**) Quantitative analysis of the number of DAPI-stained bodies in diakinesis nuclei for the indicated genotypes. (**D**) Quantification of frequencies of male offspring among the progeny and (**E**) progeny viability of the indicated genotypes. Statistical analyses were conducted using *t***-**test, n.s., not significant *P*> 0.05; * *P*< 0.05.

Decreased CO formation in *cosa-1-4A* and *cosa-1-6A* mutants is likely due to a compromised interaction between COSA-1 and other pro-CO factors, such as MSH-5 and ZHP-3. If so, insufficient levels of COSA-1 may be recruited to CO-designated sites to promote CO formation. To test this hypothesis, we artificially tethered the interaction-defective COSA-1 proteins to ZHP-3 or MSH-5 and examined the effect on CO formation. To do so, we generated an OLLAS::COSA-1-4A::3×FLAG::GFP-nanobody fusion and crossed the corresponding strain with strains expressing GFP-tagged ZHP-3 or MSH-5. GFP nanobodies specifically bind to GFP (59). This way, COSA-1-4A can be targeted to GFP-tagged ZHP-3 or MSH-5 via the fused GFP nanobody (Figure 6A). In contrast to the dim COSA-1 foci and the delayed compaction of ZHP-3 observed in *OLLAS::cosa-1-4A* mutants (Figures 3E and 4C), bright COSA-1 and ZHP-3 foci were detected in the pachytene nuclei of strains carrying *OLLAS::cosa-1-4A::3×FLAG::GFP-nanobody; zhp-3::GFP* and *OLLAS::cosa-1-4A::3×FLAG::GFP-nanobody; GFP::msh-5* (Figure 6A). These bright COSA-1 foci colocalized with the GFP-tagged ZHP-3 and MSH-5 foci, indicating that the GFP nanobody successfully directed the COSA-1-4A to the CO-designated sites (Figure 6A). Although COSA-1-4A could be recruited to the CO-designated sites by artificially tethering to MSH-5, we found an increased number of DAPI-stained bodies in *OLLAS::cosa-1-4A::3×FLAG::GFP-nanobody; GFP::msh-5* strains (10.7, *N*= 18, *P*<0.01), compared with those in *OLLAS::cosa-1-4A::3×FLAG::GFP-nanobody*worms (9.5, *N*= 21)(Figure 6B, C), suggesting that the function of MSH-5 may be compromised by this nanobody-mediated interaction. This conclusion is further confirmed by *OLLAS::cosa-1::3×FLAG::GFP-nanobody; GFP::msh-5* strain, which displayed an increased number of DAPI-stained bodies (11.2, *N* = 33) (Supplementary Figure S12A, B). In contrast, *OLLAS::cosa-1::3×FLAG::GFP-nanobody; zhp-3::GFP* and *OLLAS::cosa-1::3×FLAG::GFP-nanobody* strains showed six DAPI-stained bodies (Supplementary Figure S12A, B). Notably, we observed increased bivalent formation in the oocytes of *OLLAS::cosa-1-4A::3×FLAG::GFP-nanobody; zhp-3::GFP* strains (Figure 6B, C). The average number of DAPI-stained bodies was 7.6 (*N* = 36), compared with 9.5 (*N* =21, *P*<0.0001) in *OLLAS::cosa-1-4A::3×FLAG::GFP-nanobody* worms. Careful examination of spread nuclei in late pachytene revealed that the percentage of bubble-like structure at the crossover designation sites was increased in *OLLAS::cosa-1-4A::3×FLAG::GFP-nanobody; zhp-3::GFP* strains (25.4%, compared with 19.1% in *cosa-1-4A* mutants), indicating that the GFP-nanobody mediated interaction between COSA-1-4A and ZHP-3 partially restored the CO formation in *cosa-1-4A* mutants (Figure 5E). Indeed, the Him phenotype, which is attributed to a defective CO formation, was also alleviated in *OLLAS::cosa-1-4A::3×FLAG::GFP-nanobody; zhp-3::GFP* worms (Figure 6D). However, the progeny viability of the *OLLAS::cosa-1-4A::3×FLAG::GFP-nanobody; zhp-3::GFP* strain was not increased (Figure 6E). Cytological analyses of this strain revealed that although the bivalent differentiation is normal (Supplementary Figure S12C), 32% of late pachytene nuclei (*N*= 89) in *OLLAS::cosa-1-4A::3×FLAG::GFP-nanobody; zhp-3::GFP* worms had more than 6 ZHP-3 foci, indicative of excess CO designation (Supplementary Figure S12D).

To genetically examine whether increased recombination takes place in the presence of excess CO designation, we employed fast genetic mapping using insertion-deletion polymorphism between N2 and Hawaii chromosomes (49). The *OLLAS::cosa-1-4A::3×FLAG::GFP-nanobody* strain showed a reduced recombination rate on chromosome II (26.4% compared with 41.7 in wild type) (Supplementary Figure S12E). Consistent with increased bivalent formation, the CO frequency was restored in *OLLAS::cosa-1-4A::3×FLAG::GFP-nanobody; zhp-3::GFP*. In addition, double COs (5.26%) could be detected in *OLLAS::cosa-1-4A::3×FLAG::GFP-nanobody; zhp-3::GFP*. However, double COs (1.43%) could also be detected in *OLLAS::cosa-1-4A::3×FLAG:: GFP-nanobody* mutant, which has no excess CO designation, suggesting that CO designation and formation are separable events. Taken together, our data suggest that the congression of COSA-1 foci is facilitated by its N-terminal interaction domain. Tethering COSA-1 to ZHP-3 largely bypasses the CO defect associated with N-terminally mutated COSA-1, highlighting that the interaction between COSA-1 and ZHP-3 is required for CO formation.

### Depletion of RTEL-1 partially restores bivalent formation in *cosa-1-4A* mutants

**Figure 7.**
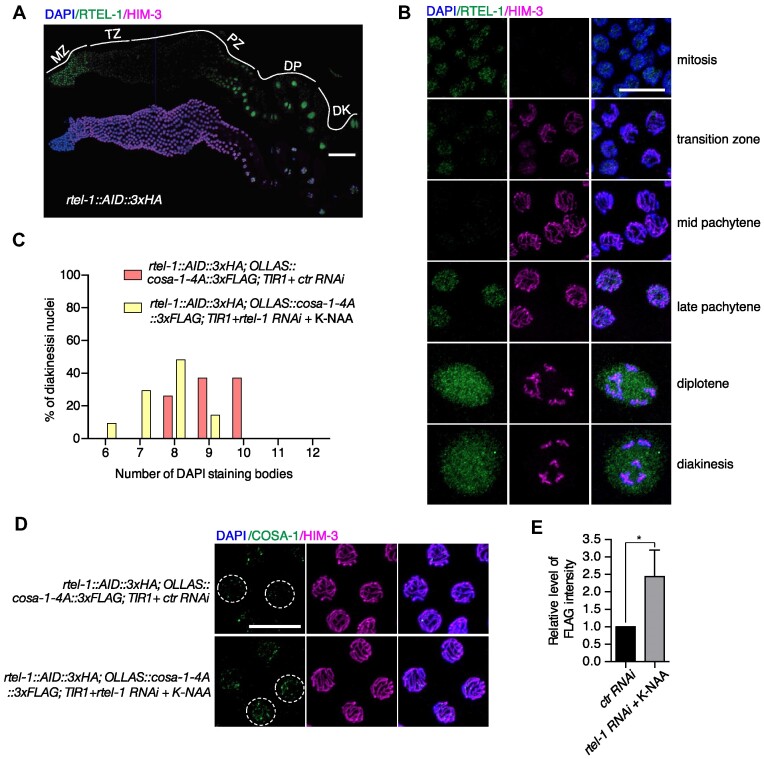
Depletion of RTEL-1 partially restored bivalent formation in *cosa-1-4A* mutants. (**A**) Whole gonad immunostaining with antibodies against HA to detect RTEL-1 (green) and HIM-3 (magenta). Scalebar, 50 μm. (**B**) Representative images of nuclei from indicated regions of germline stained with antibodies against HA to detect RTEL-1 (green) and HIM-3 (magenta). Scalebar, 10 μm. (**C**) Quantification of the number of DAPI-stained bodies in diakinesis nuclei for the indicated genotypes. (**D**) Representative images of late pachytene nuclei from control and RTEL-1 depleted germline stained with antibodies against FLAG to detect COSA-1 (green) and HIM-3 (magenta), counterstained with DAPI (blue). Scalebar, 10 μm. (**E**) Quantification of the FLAG signal (COSA-1) in nuclei from control and RTEL-1 depleted germline. Statistical analyses were conducted using *t***-**test, * *P*< 0.05.

One current view for the function of pro-CO proteins is that these proteins can protect CO-designated late-stage recombination intermediates from non-CO (NCO) activities. Compromised accumulation of pro-CO proteins at the CO-designated sites could expose CO intermediates to anti-recombinases, such as RTEL-1. We thus exploited the auxin-inducible degradation (AID) system to test whether the RTEL-1 anti-recombinase caused the univalent formation in *cosa-1-4A* mutants. We tagged the endogenous RTEL-1 with AID and three tandem HA epitopes using CRISPR-mediated genome editing. In contrast to the *rtel-1* mutants, which display a reduced brood size and retarded life cycle (60), this worm strain showed no evident defect in progeny viability, brood size, and the development of offspring, indicating that the AID tag doesn’t interfere with the function of RTEL-1 (Supplementary Figure S13A-C). The expression and localization of RTEL-1 in the germline were examined as a comprehensive localization of RTEL-1 in the germline has yet to be reported. RTEL-1 displays nuclear staining in the mitotic zone with a dramatic drop at meiotic entry (Figure 7A, B). RTEL-1 was hardly detected in mid-pachytene but reappeared as a diffuse nucleoplasmic signal in late pachytene and persisted through diplotene and diakinesis (Figure 7A, B).

The expression of RTEL-1 became undetectable throughout the germline after treatment with potassium 1-naphthylacetate (K-NAA), indicating that RTEL-1 was efficiently depleted (Supplementary Figure S13D). As previously reported, depletion of RTEL-1 resulted in a developmental delay of the F1 generation (Supplementary Figure S13E) (60). Intriguingly, we found that depletion of RTEL-1 partially restored bivalent formation in *cosa-1-4A* mutants, as revealed by increased chiasma formation (Supplementary Figure S13F) and a decreased number of DAPI-stained bodies (from 9.11 to an average of 7.67, *P*< 0.0001) (Figure 7C). Furthermore, localization of COSA-1 also appeared to be partially rescued by RTEL-1 depletion: more COSA-1 signals could be detected on chromosomes after depletion of RTEL-1 in late pachytene (Figure 7D). The signal intensity of COSA-1 was significantly stronger (2.5×), and some nuclei even contained bright COSA-1 foci (Figure 7D, E). However, we hardly detected COSA-1 signal in early pachytene in both control RNAi and RTEL-1 depletion strains (Supplementary Figure S13G, H). The intensity of COSA-1 signal showed a slight increase (1.46×) in mid pachytene after depletion of RTEL-1, indicating that RTEL-1 mainly function in late prophase (Supplementary Figure S13G, I). Together, these data suggest that the COSA-1 mediated pro-CO complex formation or stabilization may safeguard the CO-designated recombination intermediates from disassembly by the RTEL-1 helicase.

### *cosa-1-4A* mutants exhibit an increased number of univalent in the presence of unresolved meiotic recombination intermediates

**Figure 8.**
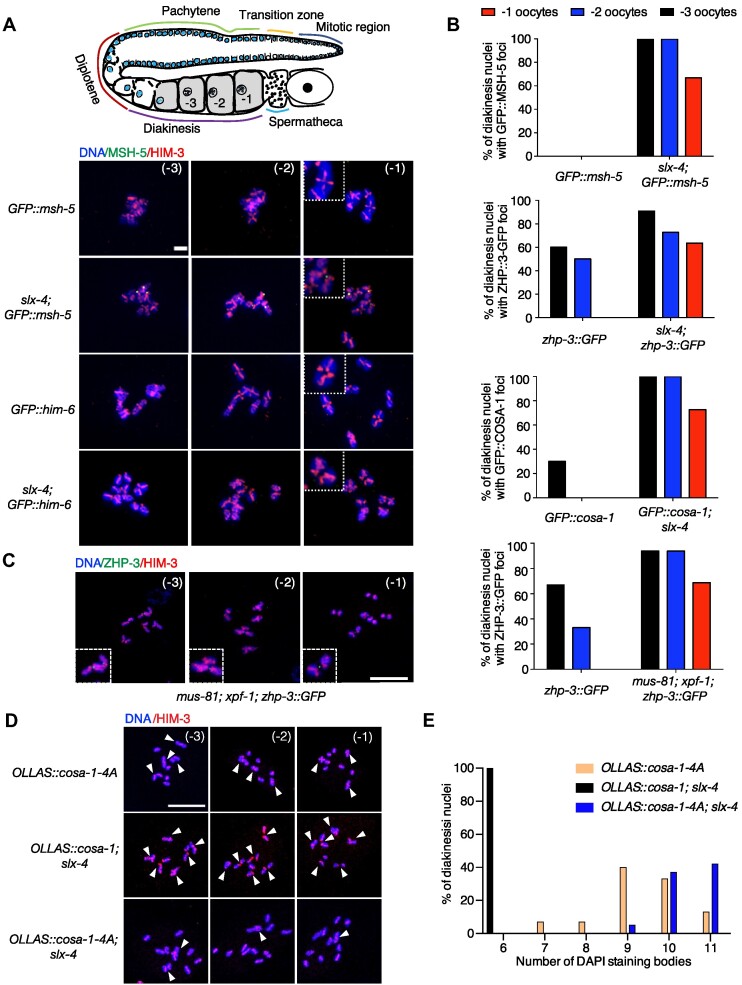
*cosa-1-4A* mutants exhibited more univalents in the presence of unresolved meiotic recombination intermediates. (**A**) Representative images of the three last oocytes in diakinesis of the indicated genotype. Persistent GFP::MSH-5 foci (green) localized between two univalent pairs. Scalebar, 5 μm. (**B**) Quantification of foci of indicated pro-CO proteins in late diakinesis oocytes from wild-type and *slx-4* mutants. (**C**) Representative images of the three last oocytes in diakinesis of *mus-81; xpf-1* mutants. Persistent ZHP-3::GFP foci (green) localized between two univalent pairs. Scalebar, 10 μm. (**D**) Representative images of the three last oocytes in diakinesis of the indicated genotype. The nuclei were stained with antibodies against HIM-3 (red), counterstained with DAPI (blue). Scalebar, 10 μm. White arrowheads indicate bivalents or dissociated bivalents. (**E**) Quantification of the number of DAPI-stained bodies in diakinesis nuclei for the indicated genotypes.

During meiosis I prophase, bright COSA-1 foci are initially detected in late pachytene and gradually disappear as nuclei progress into diakinesis (26). Previous studies showed that unresolved recombination intermediates caused by *slx-4* mutation led to delayed dissociation of COSA-1 or ZHP-3 from CO-designated sites in diakinesis oocytes (38,61). Indeed, no ZHP-3, COSA-1 and MSH-5 foci could be detected in –1 oocytes in wild type, although residual foci occurred in –2 and –3 oocytes (Figure 8A, B). In contrast, more than 60% of –1 oocytes contained COSA-1, ZHP-3, and MSH-5 foci in *slx-4* mutants (Figure 8A, B). These foci colocalized with the junction of ‘univalent pairs’, which are also referred to as dissociated bivalents (Supplementary Figure S14A, C). To further confirm that the persistent COSA-1, ZHP-3 and MSH-5 foci were caused by unresolved recombination intermediates, we checked the ZHP-3 foci in *mus-81; xpf-1* nuclease defective double mutants, in which both pathways for recombination intermediate resolution are compromised (62). Similar to *slx-4* mutants, 69% of –1 oocytes in *mus-81; xpf-1* double mutants had persistent ZHP-3 foci (Figure 8C). The persistent foci of pro-CO proteins were not due to excessive foci formation, as only six CO-designated sites marked by COSA-1, ZHP-3 or MSH-5 could be observed at late pachytene in *slx-4* mutants (Supplementary Figure S14B, D). Interestingly, we couldn’t observe any HIM-6 foci in diakinesis oocytes of *slx-4* mutants (Figure 8A). Similar to the wild type, the HIM-6 foci disappeared at diplotene and redistributed throughout the nucleoplasm towards the end of prophase I in *slx-4* mutants, suggesting that the dissociation of HIM-6 from the homologous chromosomes does not require the resolution of recombination intermediates.

What is the biological function of those persistent pro-CO proteins' foci in *slx-4* mutant nematodes? We hypothesized that persistent COSA-1 or ZHP-3 foci at the junction of ‘univalent pairs’ stabilize unresolved recombination intermediates and prevent ‘univalent pairs’ from premature dissociation. Based on this hypothesis, failure of COSA-1 or ZHP-3 foci formation should lead to increased univalent formation in *slx-4* mutants. However, deletion of *cosa-1* or *zhp-3* usually results in very strong phenotypes as revealed by no CO designation and formation of 12 univalents, therefore hindering further analysis of their function in the stabilization of the unresolved recombination intermediates. We, therefore, addressed this issue by using the *cosa-1-4A* hypomorphic allele and found that *cosa-1-4A; slx-4* double mutants displayed more DAPI-stained bodies than *slx-4* and *cosa-1-4A* single mutants in diakinesis oocytes (from 9.4 on average to 10.79, *P*< 0.001) (Figure 8D, E), suggesting that univalent pairs linked by the unresolved recombination intermediates may become unstable without protection by pro-CO proteins. Interestingly, chromosome remodeling into CO distal and proximal domains happened normally in *cosa-1-4A; slx-4* double mutants (Supplementary Figure S15), further enforcing the notion that COSA-1 protects or stabilizes late recombination intermediates destined to be resolved by SLX-4 mediated endonucleases.

## Discussion

### Pro-CO complex assembly via interactions among pro-CO factors

The co-existence of COSA-1 orthologs and the MSH4-MSH-5 complex in Metazoa except in the *Drosophila* genus, together with their roles in CO formation, suggests that COSA-1 and MSH4-MSH-5 may act as a functional module (26). Indeed, COSA-1, MSH-5, and ZHP-3 colocalized at the CO-designated sites and displayed interdependence for their localization, indicating that they may form a pro-CO complex at CO-designated sites (26). So far, no physical interaction among those pro-CO proteins has been reported. In this study, we investigated the interaction among pro-CO proteins using TurboID and yeast two-hybrid assays. We found that COSA-1 contains an intrinsically disordered region at the N-terminus, which interacts with MSH-5 and ZHP-3. Intrinsically disordered regions are frequently involved in protein-protein interaction and act as hubs in protein interaction networks (63). Indeed, deletion of the N-terminal intrinsically disordered region of COSA-1 abolished its interaction with MSH-5 and ZHP-3, suggesting that this region is important for pro-CO complex formation. Although sequence alignment revealed a high variability both in length and sequence of the N-terminus of COSA-1 orthologs, a short isoform of mouse CNTD1, which lacks the first 85 amino acids also fails to interact with key factors for CO formation, such as MSH4, MSH5 and CDK2 (53). These findings indicate a conserved role of the N-terminus of COSA-1 orthologs in the interaction with other pro-CO factors. In mice testis, the short form of CNTD1 is predominant and hypothesized to regulate COs via the interaction with the replication factor C complex (53). It will be interesting to investigate whether the full-length form of CNTD1 exists in female germ cells and whether CO formation requires different CNTD1 isoforms associated with gender-specific partners.

HIM-6 has been reported to play a role in promoting meiotic COs, and it also accumulates at the CO-designated sites (31,52,64). During late pachytene, it is detected as a doublet at CO sites with COSA-1 localized at the center. However, we didn’t detect interactions between HIM-6 and COSA-1, or any other pro-CO factors, such as ZHP-3 and MSH-5. Unlike other pro-CO factors, the dissociation of HIM-6 foci from CO-designated sites was not delayed by unresolved recombination intermediates. In addition, *him-6* mutants are proficient for loading MutSγ and CO designation (35,64,65), indicating that HIM-6 might not be a core component of the pro-CO complex. These results reinforce the idea that HIM-6 is more likely to play a pro-CO role late in the CO pathway by enforcing biased resolution of recombination intermediates into COs (64), rather than function at an early stage of CO recombination by forming a complex with other pro-CO proteins.

### CO formation requires assembly and accumulation of pro-CO factors at CO-designated sites

We observed that point mutations located in the N-terminus of COSA-1 impair the interaction with ZHP-3 and MSH-5 and compromise the accumulation of COSA-1 at CO-designated sites (Figure 3E). However, in contrast to the previously reported *cosa-1* mutants, in which MSH-5 foci could not be detected (26), MSH-5 still appears as six bright foci in the late pachytene nuclei from these interaction-compromised *cosa-1* mutants as in wild-type (Figure 4A, B). In addition, ZHP-3 eventually became restricted at CO-designated sites although a bit delayed and the chromosome remodeling occurred normally (Figure 4C, D and Supplementary Figure S10). These observations suggest that CO could be designated without distinct COSA-1 foci formation.

Although CO designation seems to occur normally in the interaction-compromised *cosa-1* mutants, CO formation is severely compromised as revealed by frequent univalent formation in diakinesis oocytes. We speculated that, without COSA-1 accumulation at the CO-designated sites, those distinct MSH-5 or ZHP-3 foci in *cosa-1-4A* mutants are not sufficient for CO formation, even though the CO designation is successful. A previous study suggested a self-reinforcing model for CO formation, in which both enrichment of pro-CO factors at CO-designated sites and their depletion from sites elsewhere on the chromosome can be achieved by COSA-1 associated with the CDK-2 (cyclin-dependent kinase 2) (26). Consistent with this model, a recent finding showed that MSH-5 can be phosphorylated by COSA-1/CDK-2 (40). This phosphorylation promotes the stable association of COSA-1/CDK-2 to the CO-designated sites and generates positive feedback for assembly of high-order pro-CO complexes, also known as ‘recombination nodules’ (66,67). We found that COSA-1-4A retained the ability to interact with CDK-2, therefore it may still be able to phosphorylate MSH-5 via weak interactions between COSA-1-4A and MSH-5 or ZHP-3. The phosphorylation of MSH-5 could happen either at the CO-designated sites due to the residue COSA-1-CDK-2 at those sites or before their colocalization to the CO-designated sites, which is supported by the appearance of MSH-5 p1009T antibody staining in early pachytene. While this phosphorylation may be sufficient for the enrichment of MSH-5 and restriction of ZHP-3 at the CO-designated sites, it is probably not enough for the formation of a fully functional ‘recombination nodule’ if the accumulation of COSA-1 is compromised. Consistent with this notion, artificially tethering interaction-defective COSA-1(COSA-1-4A) to ZHP-3 restored the distinct COSA-1 foci formation at the CO-designated sites and partially rescued the defective CO formation, suggesting that accumulation of COSA-1 into distinct foci is required for formation or stabilization of functional pro-CO complexes.

Taken together, our results indicate that CO designation and formation are separable events and that CO designation is necessary but not sufficient for CO formation. This conclusion is also supported by previous studies on HJ resolution mutants, which display successful CO designation marked by six distinct pro-CO protein foci and the reciprocal localization of SYP-1 and HTP-1/2 while showing dramatically decreased CO frequency (35,37).

### Pro-CO complex promotes CO formation by protecting recombination intermediates from being dismantled prematurely by the RTEL-1 anti-recombinase

An outstanding question that remains is the function of the pro-CO complex at CO-designated sites. Pro-CO proteins have long been assumed to be able to stabilize recombination intermediates to promote CO formation. Careful examination of the dynamic organization of pro-CO proteins at the CO-designated sites and their relationship to the synaptonemal complex central region (SC-CR) suggests that the concentrated pro-CO complex enveloped by SC-CR is important for CO maturation by generating a CO-specific repair compartment (31,67). This unique compartment may protect the CO intermediates from NCO activities. Here, we revealed that the CO formation defect observed in the interaction-compromised *cosa-1* mutants could be partially bypassed by depletion of the RTEL-1 helicase, suggesting that the COSA-1 mediated pro-CO complex formation or stabilization could also protect CO-designated meiotic recombination intermediates from disassembly by anti-recombinases, such as RTEL-1. RTEL-1 can efficiently dismantle D-loop recombination intermediates to limit excessive CO formation (68). Therefore, CO-designated sites are very likely to be the sites where D-loops transit into HJs. We found that RTEL-1 could be detected in the mitotic zone but disappears as soon as cells enter meiosis. The signal of RTEL-1 reappeared in late pachytene where the CO or NCO outcomes are visibly manifested and persisted through diplotene and diakinesis, indicating a role of RTEL-1 at late stages of meiotic prophase I. A recent study showed that SPO-11-mediated DSBs continuously form until mid-late pachytene during meiotic progression in *C. elegans* (69). DSBs formed at late stages are essential for CO formation. It is possible that RTEL-1 acts on the D-loop recombination intermediates arising from late DSBs and promotes NCO repair if the recombination intermediates are not protected by pro-CO complexes.

We confirmed that unresolved meiotic recombination intermediates caused by mutation of *slx-4* or HJ resolvases resulted in persistent CO-designated sites till diakinesis oocytes, suggesting that pro-CO complex may also protect late CO intermediates, such as HJs, until they are resolved by CO-specific resolvases (38,61). Conversely, if pro-CO complex formation is impaired and CO intermediates are not resolved timely by specific resolvases, they may be dissolved or disrupted to form NCO. Indeed, we found that the CO formation defect became more severe in the interaction-compromised *cosa-1* mutants when combined with *slx-4* mutation, as evidenced by more univalent formation. Therefore, protection or stabilization of the unresolved recombination intermediates by pro-CO complex could prevent the premature dissociation of linked ‘univalent pairs’, which might be processed by nucleases, such as LEM-3 during late meiotic stages, to avoid genome instability (61).

In summary, combined with previous studies and our data, we suggest that two structures in *C. elegans* prevent CO intermediates from being dismantled: the enveloping structure formed by the SC-CR and the pro-CO complex formed by interactions among COSA-1, ZHP-3, and MSH-5. While the SC-CR could create an environment that helps to recruit and retain pro-CO factors, the maintenance of an intact pro-CO complex via physical interactions among pro-CO factors may further enhance the stability of those proteins at CO-designated sites to promote CO maturation, especially in diplotene or diakinesis, where the SC-CR bubbles disappear. The remnants of the SC-CR structure are retained only at one side of the CO site (31). In addition, the pro-CO complex seems to protect or stabilize not only early recombination intermediates, such as D-loops, but also late recombination intermediates, like HJs.

## Supplementary Material

gkae130_Supplemental_File

## Data Availability

All data generated or analyzed during this study are included in this article and its supplementary data.
