# Supplementary material for: COSA-1 mediated pro-crossover complex formation promotes meiotic crossing over in C. elegans
Source: Nucleic Acids Res. 2024 Feb 27;52(8):4375–92. doi: 10.1093/nar/gkae130 (PMC11077092; doi:10.1093/nar/gkae130)
Supplement: gkae130_Supplemental_File [file gkae130_supplemental_file.pdf]

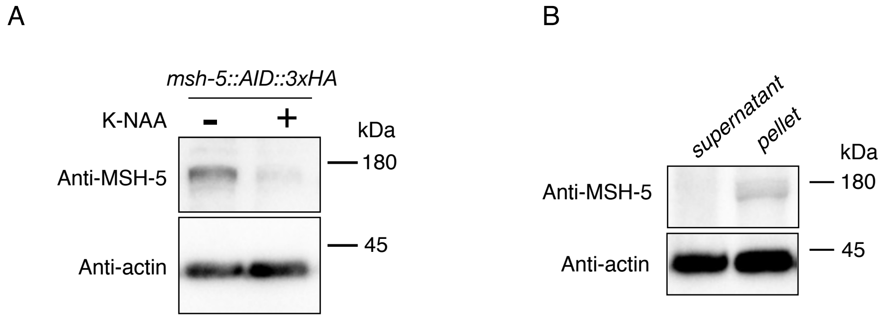

Figure S1

MSH-5 protein was barely soluble in the lysis buffer normally used for co-IP

(A) Test of MSH-5 antibodies by Western blot. (B) Analysis of solubility of endogenous MSH-5 protein.

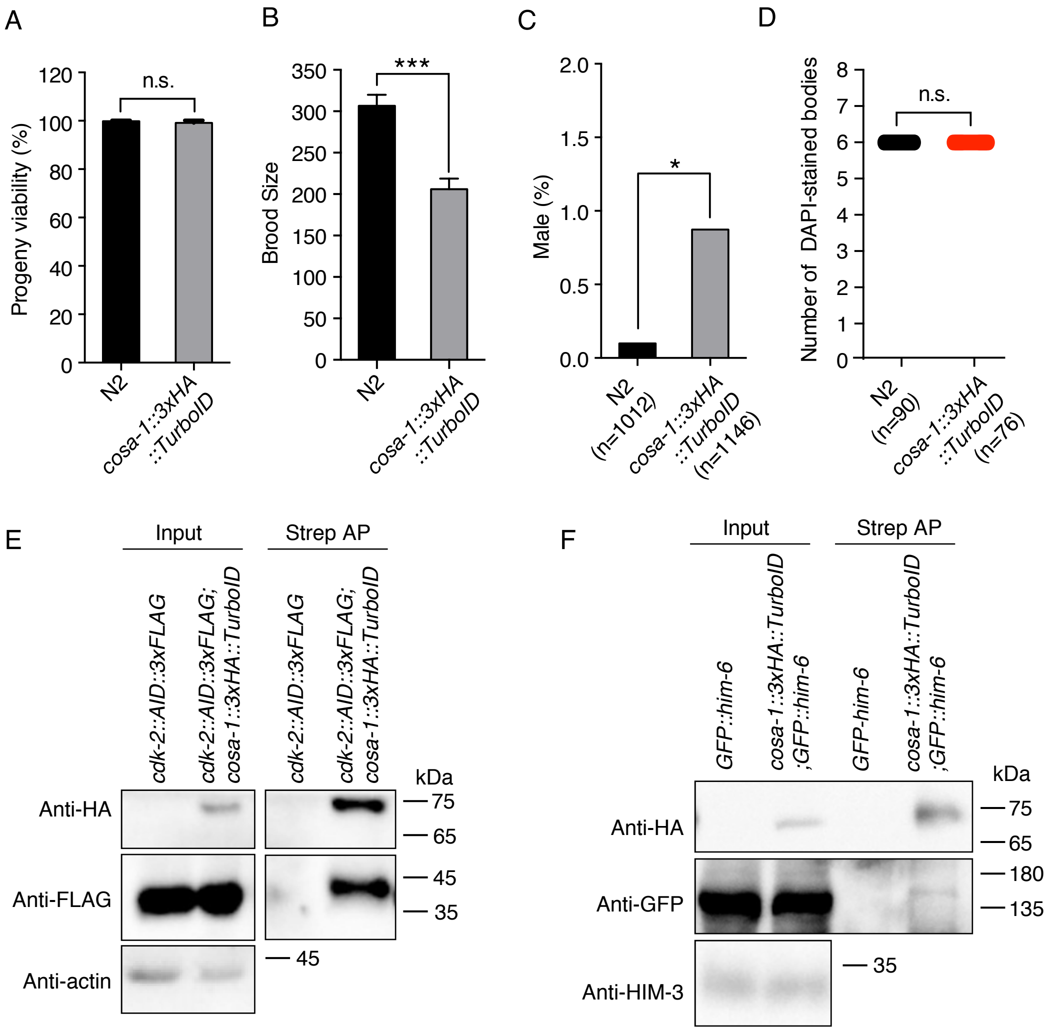

Figure S2

(A-C) Quantification of the progeny viability, brood size, and the frequency of male offspring among the progeny of *cosa-1::3xHA::TurboID* knock-in worms. Statistical analyses were conducted using T-test (A and B) and Z-test for 2 population proportions (C), n.s., not significant $p>0.05$; * $p<0.05$. (D) Quantification of the number of DAPI-stained bodies in diakinesis oocytes of N2 and *cosa-1::3xHA::TurboID* strain. (E) Western blotting analysis revealed that CDK-2 proteins are biotinylated among proteins enriched with streptavidin beads. (F) Biotinylated GFP::HIM-6 proteins are detected by western blotting with antibodies against GFP. Proteins biotinylated by TurboID were enriched with streptavidin beads and examined by western blotting.

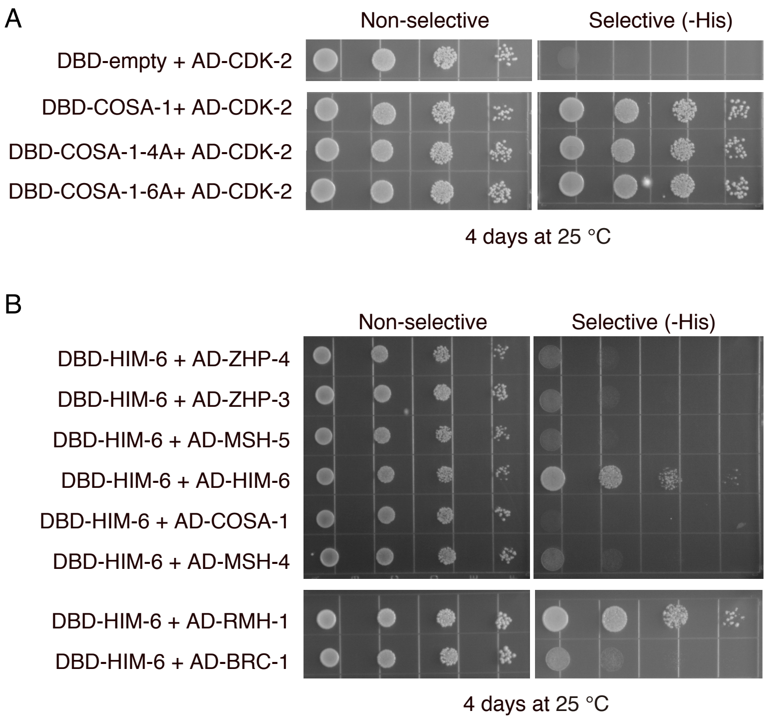

Figure S3

(A) Analysis of interactions between COSA-1 mutants and CDK-2 by yeast two-hybrid system. (B) Analysis of interactions between HIM-6 and other pro-CO proteins by yeast two-hybrid system.

Yang et al Figure S4

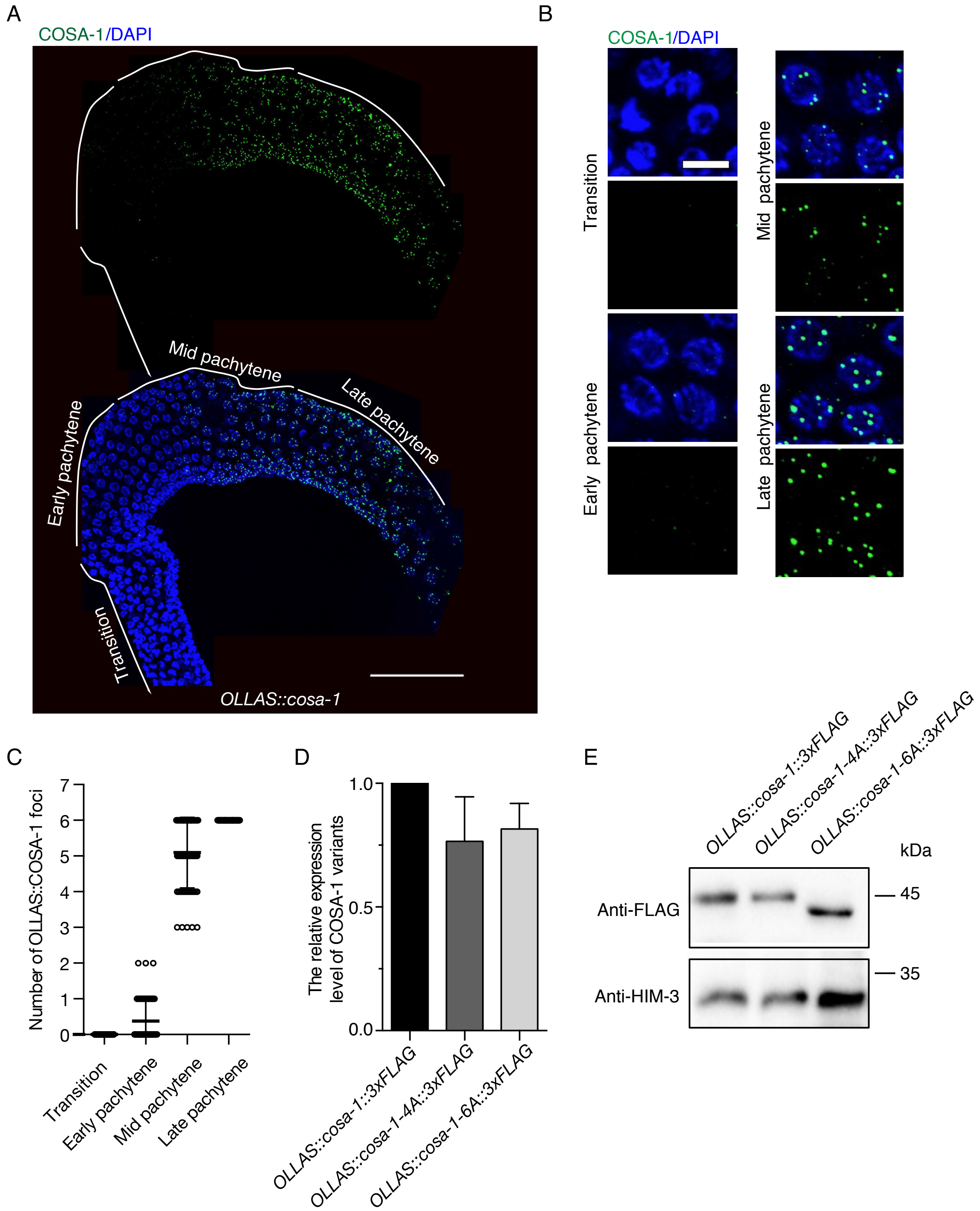

Figure S4

Analysis of interaction-compromised *cosa-1* mutants

(A) Whole gonad immunostaining with antibodies against OLLAS to detect COSA-1 (green), counterstained with DAPI (blue). Scalebar, 50 μm. (B) Representative images of nuclei from indicated regions of germline stained with antibodies against OLLAS to detect COSA-1 (green), counterstained with DAPI (blue). Scalebar, 5 μm. (C) Quantification of the OLLAS::COSA-1 foci in nuclei from indicated regions of germline. (D) Quantification of the relative expression levels of COSA-1-4A and COSA-1-6A mutant proteins compared with wild-type COSA-1. (E) Immunoblot showing the expression of the COSA-1-4A and COSA-1-6A proteins in whole worm lysates. HIM-3 was used as a loading control.

Yang et al Figure S5

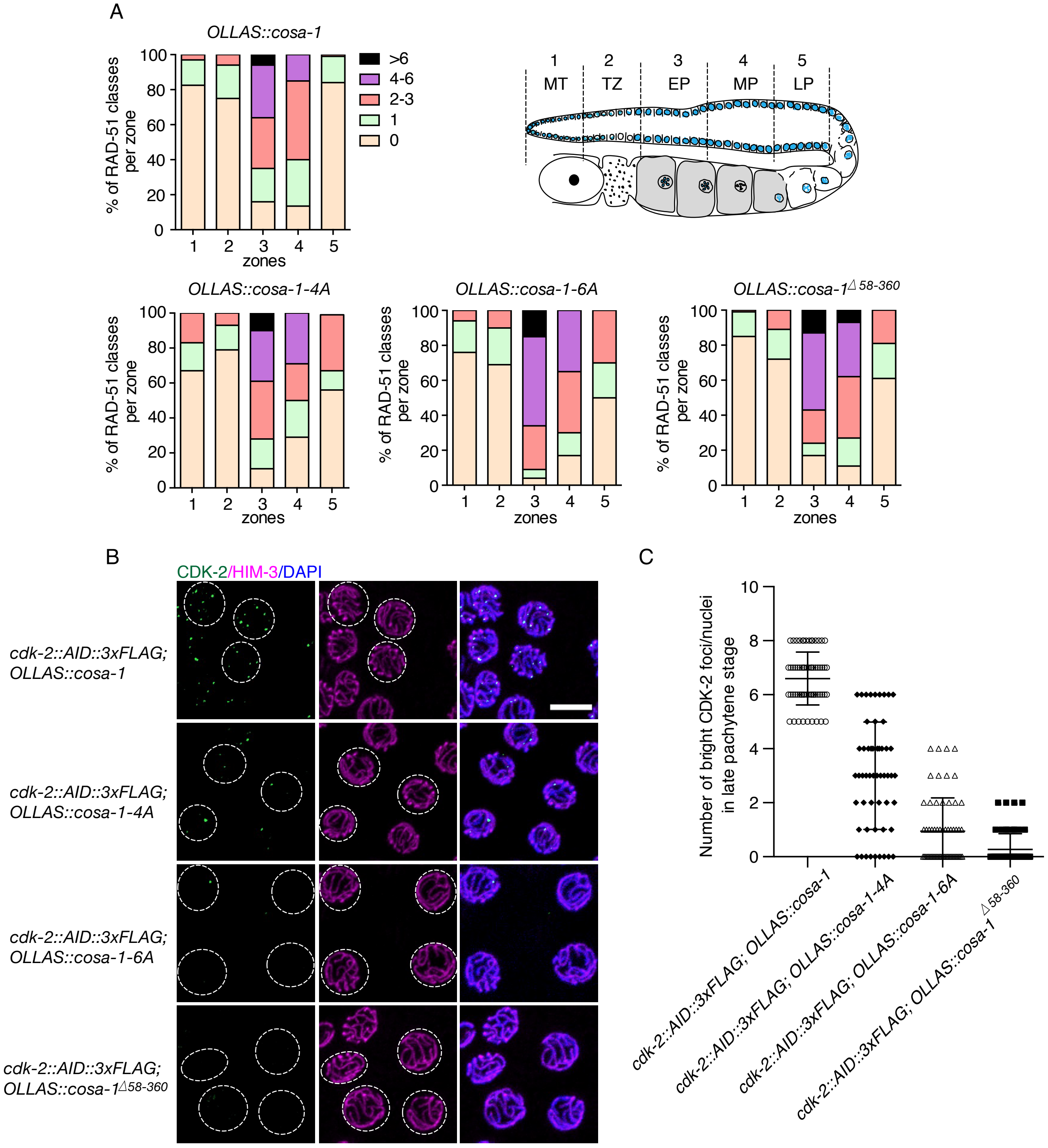

Figure S5

Analysis of RAD-51 and CDK-2 foci in *cosa-1-4A* and *cosa-1-6A* mutants

(A) Quantification of RAD-51 foci in indicated regions of the germline. Zone definitions: 1) MT, mitotic-, 2) TZ, transition-, 3) EP, early pachytene-, 4) MP, mid pachytene-, 5) LP, late pachytene zone. (B) Representative images of late pachytene nuclei of the indicated genotypes stained with antibodies against FLAG to detect CDK-2 (green) and HIM-3 (magenta), counterstained with DAPI (blue). Scalebar, 5 μm. (C) Quantification of bright CDK-2 foci in the late pachytene nuclei of *cosa-1-4A* and *cosa-1-6A* mutants.

Yang et al Figure S6

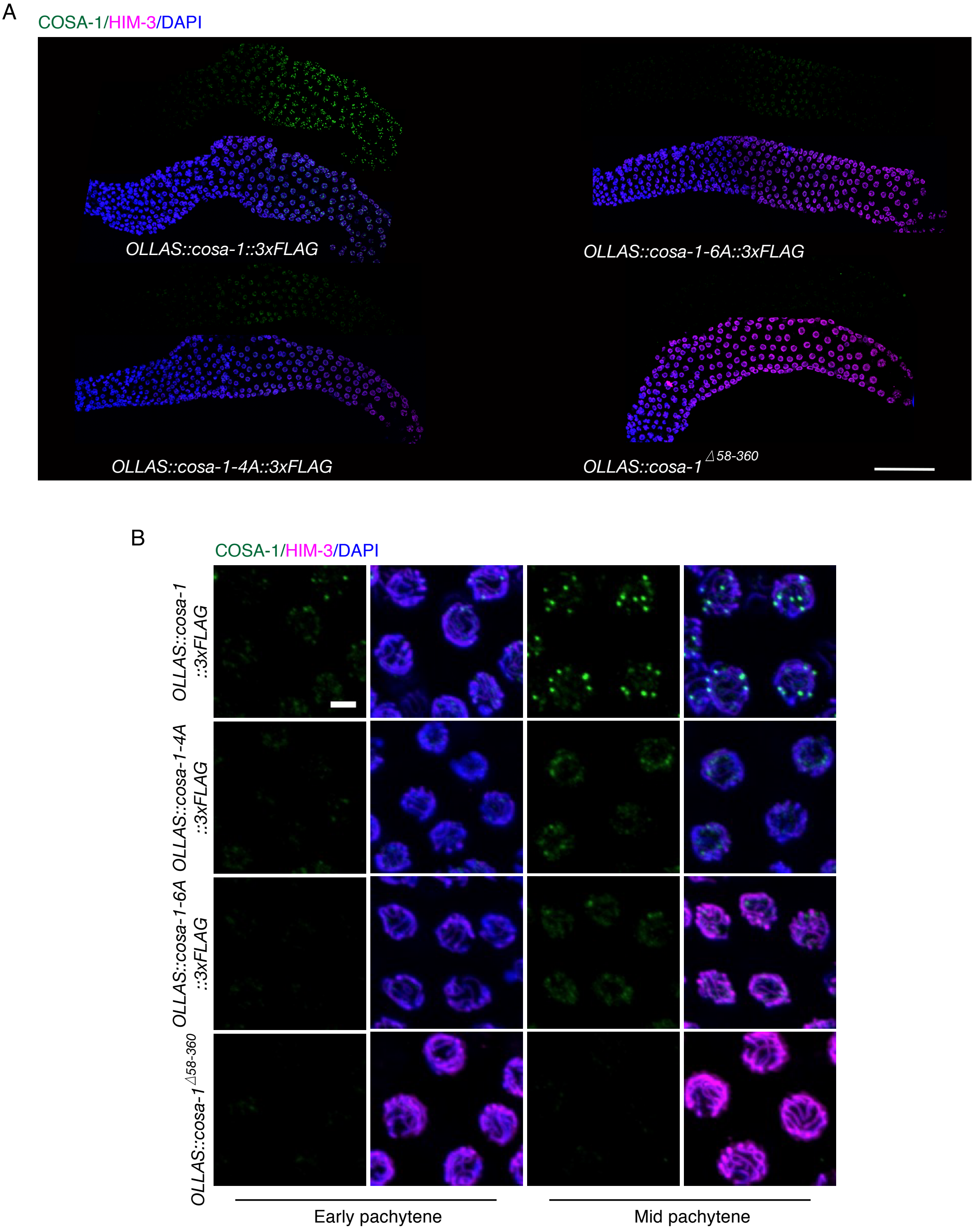

Figure S6

Analysis of COSA-1 foci in *cosa-1-4A* and *cosa-1-6A* mutants

(A) Whole gonad immunostaining with antibodies against FLAG to detect COSA-1 (green) and HIM-3 (magenta). Scalebar, 50 μm. (B)Close-up images of fields of nuclei in early and mid pachytene of the indicated genotypes. Scalebar 2 μm.

Yang et al Figure S7

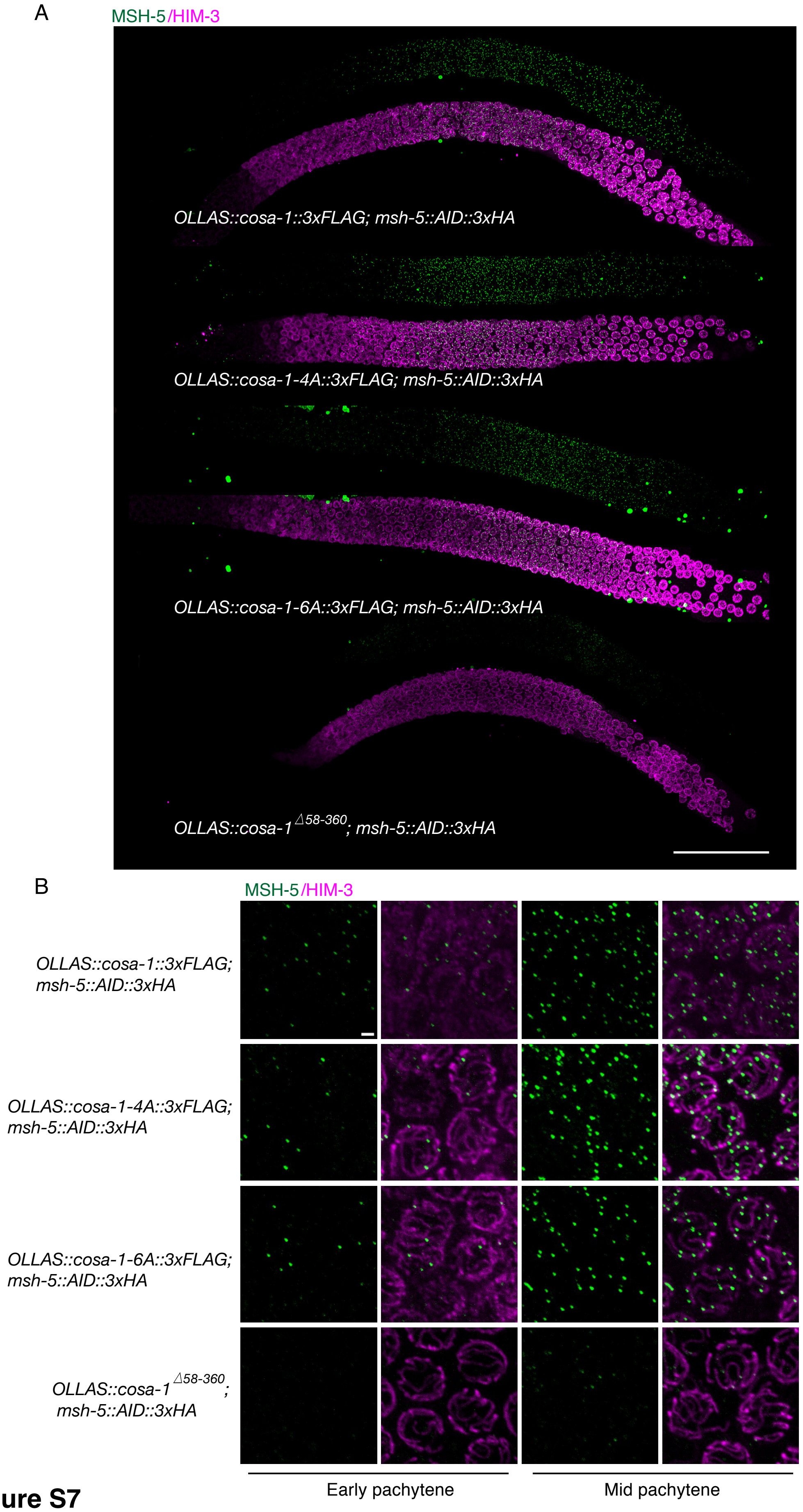

Figure S7

Localization of MSH-5 is normal in *cosa-1-4A* and *cosa-1-6A* mutants

(A) Immunostaining with antibodies against HA to detect MSH-5 (green) and HIM-3 (magenta) of spread germlines from the indicated genotypes. Scalebar, 50 μm. (B)Close-up images of fields of nuclei in early and mid pachytene of the indicated genotypes. Scalebar 2 μm.

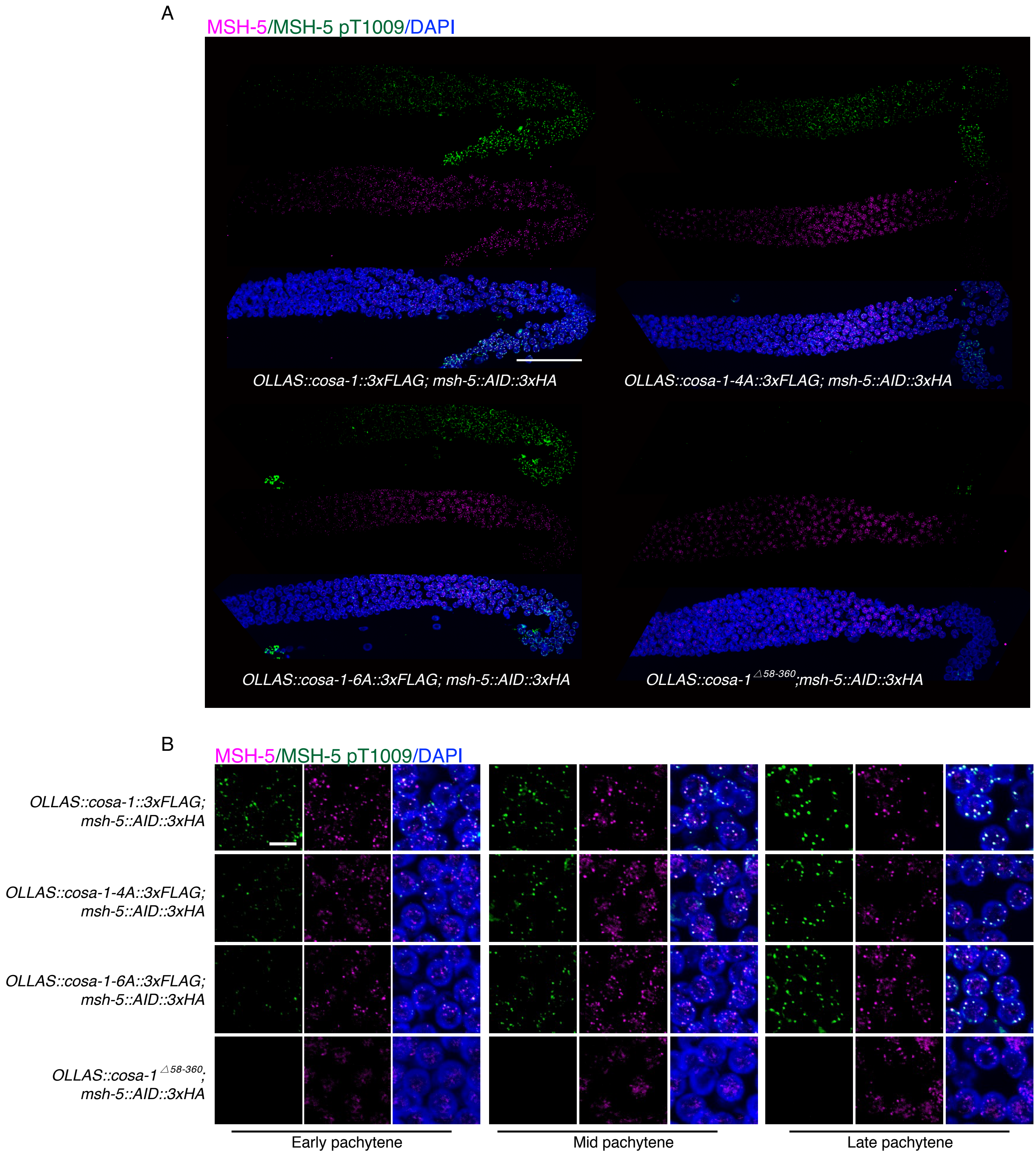

Figure S8

Phosphorylation of MSH-5 is normal in *cosa-1-4A* and *cosa-1-6A* mutants

(A) Immunostaining with antibodies against MSH-5 pT1009 (green) and MSH-5 (magenta) of spread germlines from the indicated genotypes. Scalebar, 50 μm. (B)Close-up images of fields of nuclei in early, mid and late pachytene of the indicated genotypes. Scalebar 5 μm.

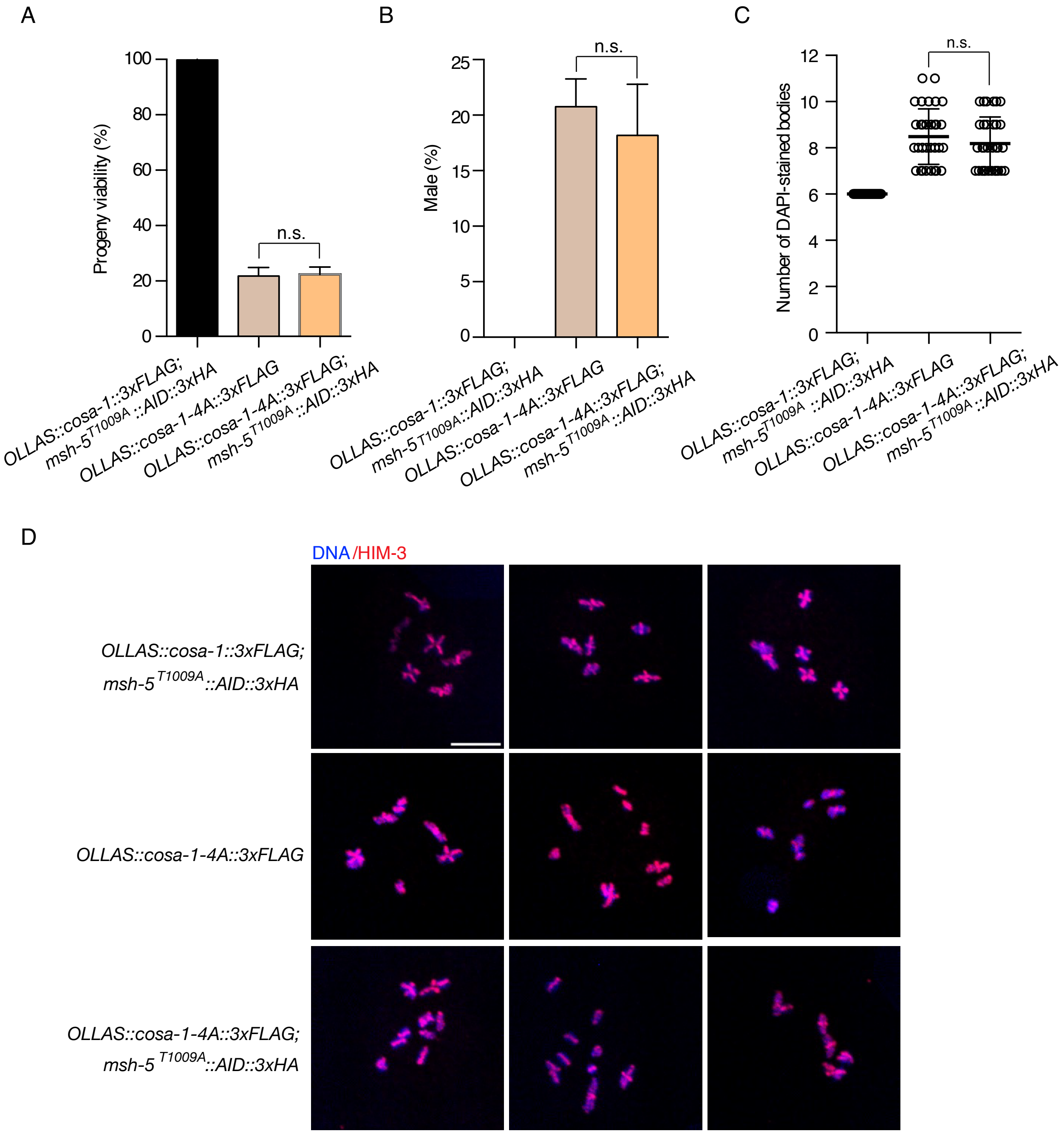

Figure S9

cosa-1-4A **didn't show synthetic phenotypes with** ***msh-5*** **phosphomutants**

(A-C) Quantification of the progeny viability, the frequency of male offspring and the number of DAPI-stained bodies in diakinesis nuclei for the indicated genotypes. Statistical analyses were conducted using T-test, n.s., not significant. (D) Representative images of diakinesis nuclei of the indicated genotypes stained with antibodies against HIM-3 (red), counterstained with DAPI (blue). Scalebar, 5 μm.

Yang et al Figure S10

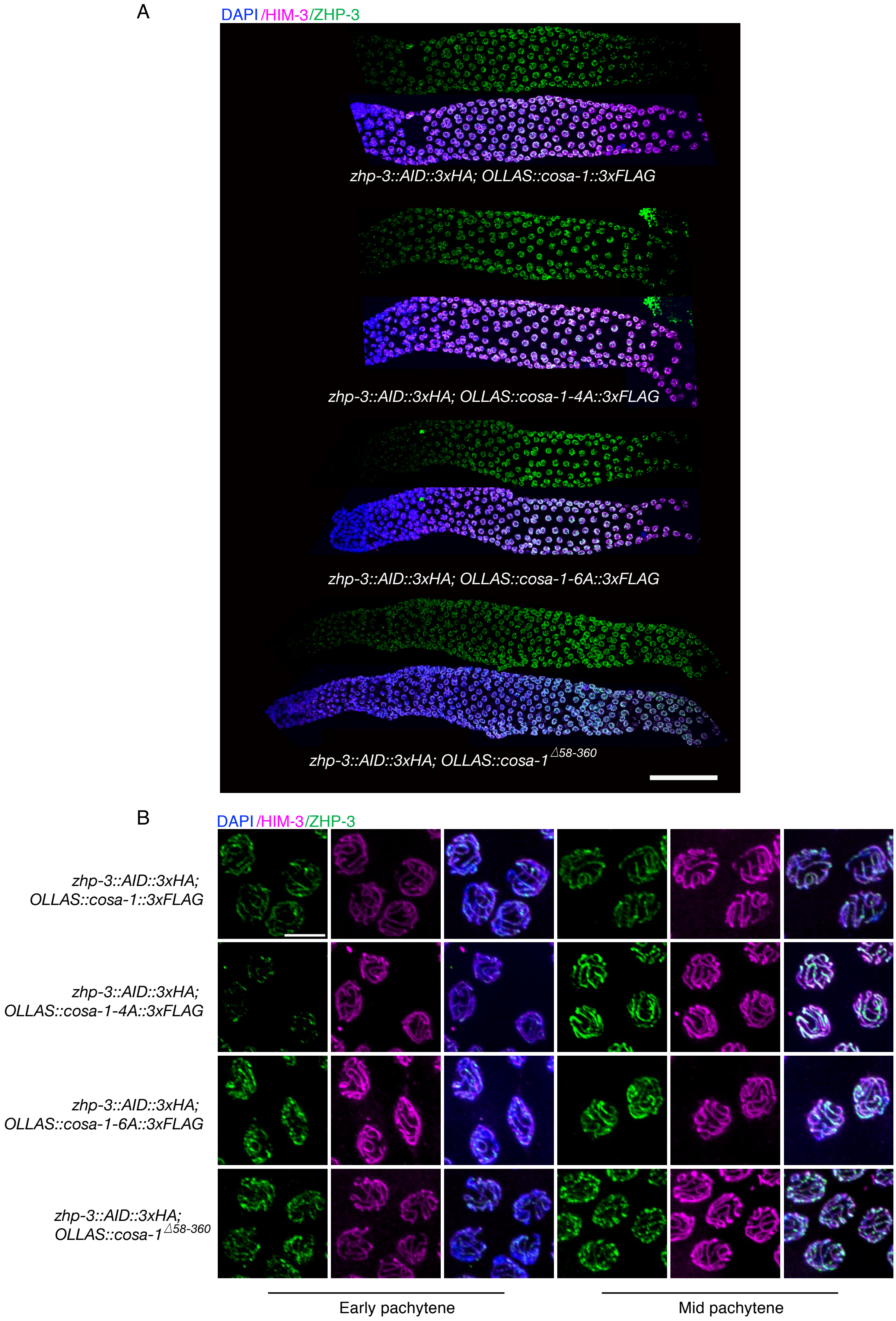

Figure S10

Delayed ZHP-3 relocalization in *cosa-1-4A* and *cosa-1-6A* mutants

(A) Whole gonad immunostaining with antibodies against HA to detect ZHP-3 (green) and HIM-3 (magenta). Scalebar, 50 μm. (B)Close-up images of fields of nuclei in early and mid pachytene of the indicated genotypes. Scalebar 5 μm.

Yang et al Figure S11

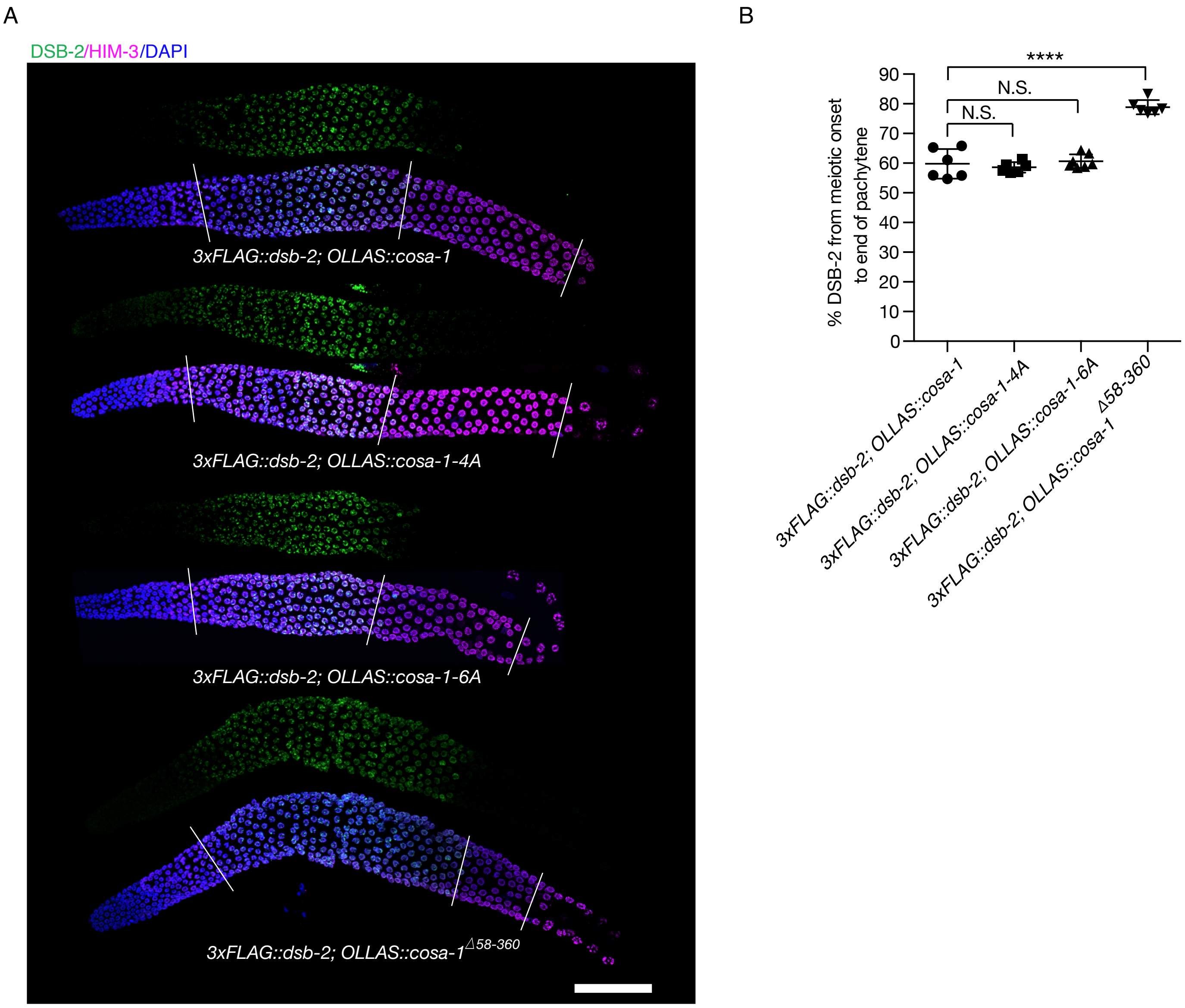

Figure S11

Localization of DSB-2 is normal in in *cosa-1-4A* and *cosa-1-6A* mutants

(A) Whole gonad immunostaining with antibodies against FLAG to detect DSB-2 (green) and HIM-3 (magenta). Scalebar, 50 μm. (B) Quantitative analysis of the DSB-2 positive zone in germlines of the indicated genotypes. The extent of the DSB-2 positive zone was defined as the percentage of the DSB-2 positive region out of the region from meiotic onset to the end of pachytene. Statistical analyses were conducted using T-test, n.s., not significant, p>0.05; **** p<0.001.

Yang et al Figure S12

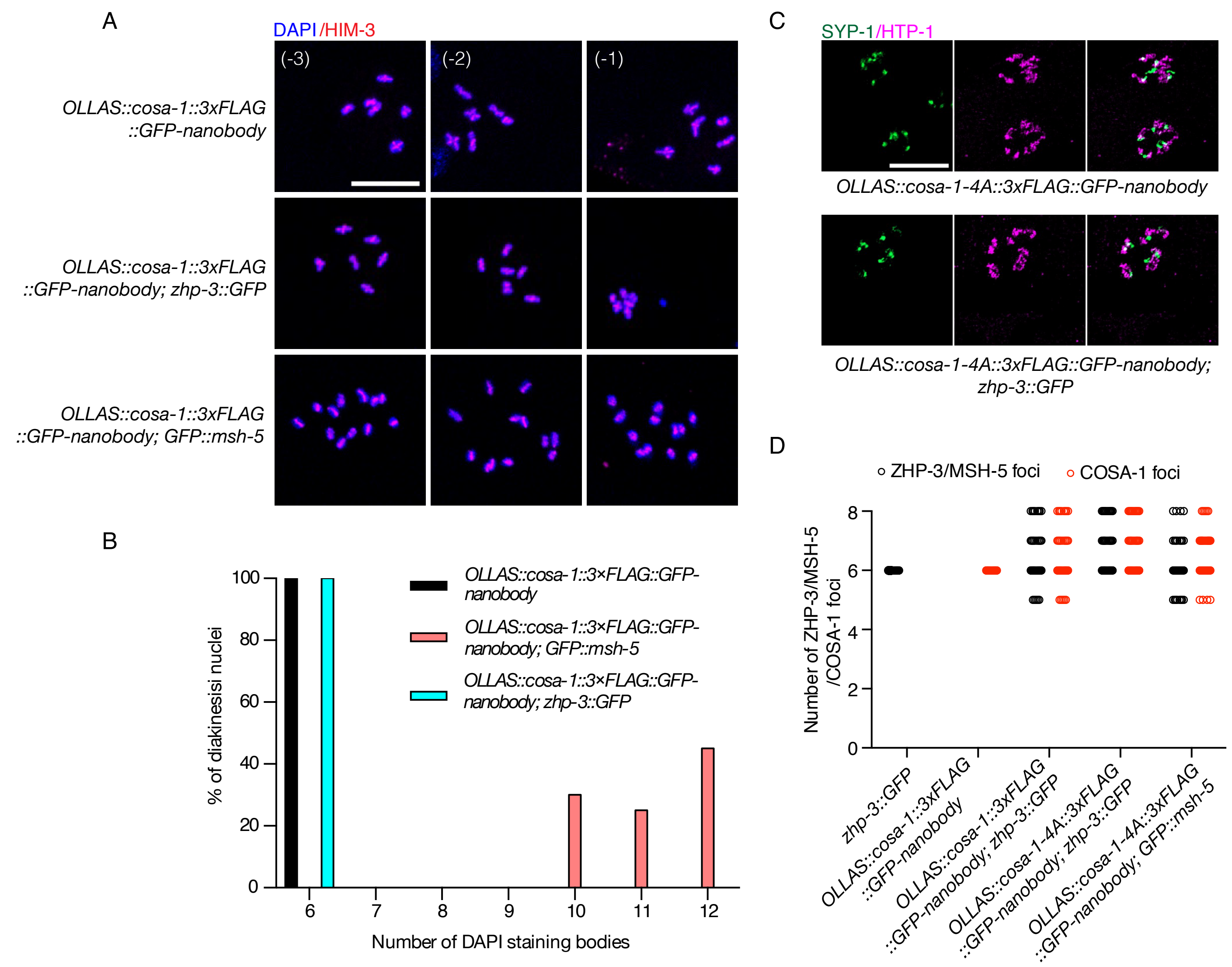

E

CO distribution and recombination frequency

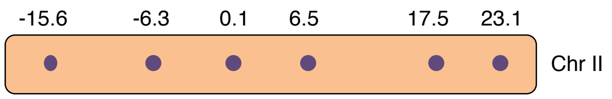

							2 CO (%)	1 CO (%)	0 CO (%)	n
Theoretical	0.093	0.064	0.064	0.11	0.056	0.387				
N2	0.158	0.086	0.065	0.108	0.043	0.417	0	58(41.73)	81(58.27)	139
zhp-3::GFP	0.147	0.068	0.063	0.089	0.068	0.389	0	74(38.95)	115(61.05)	189
OLLAS::cosa-1-4A::3 xFLAG::GFP nanobody	0.1	0.071	0.014	0.043	0.036	0.264	2(1.43)	31(22.14)	107(76.43)	140
OLLAS::cosa-1-4A::3xFLAG::GFP nanobody; zhp-3::GFP	0.116	0.116	0.095	0.158	0.042	0.527	5(5.26)	40(42.11)	40(42.11)	95

Figure S12 Artificially tethering COSA-1-4A to ZHP-3 resulted in excess CO designation
(A) Representative images of diakinesis nuclei of the indicated genotypes stained with antibodies against HIM-3 (red), counterstained with DAPI (blue). Scalebar, 5 μm. (B) Quantitative analysis of the number of DAPI-stained bodies in diakinesis nuclei for the indicated genotypes. (C) Representative images of diplotene nuclei of the indicated genotypes stained with antibodies against SYP-1 (green) and HTP-1 (magenta). Scalebar, 5 μm. (D) Quantification of COSA-1, ZHP-3 or MSH-5 foci in late pachytene nuclei of the indicated genotypes. (E) Analysis of CO frequency and distribution on chromosome II of the indicated genotypes. n is the number of cross-progeny scored. The frequency of 2 COs, 1 CO or 0 CO per chromosome is indicated in absolute numbers and as percentage (in brackets).

Yang et al Figure S13

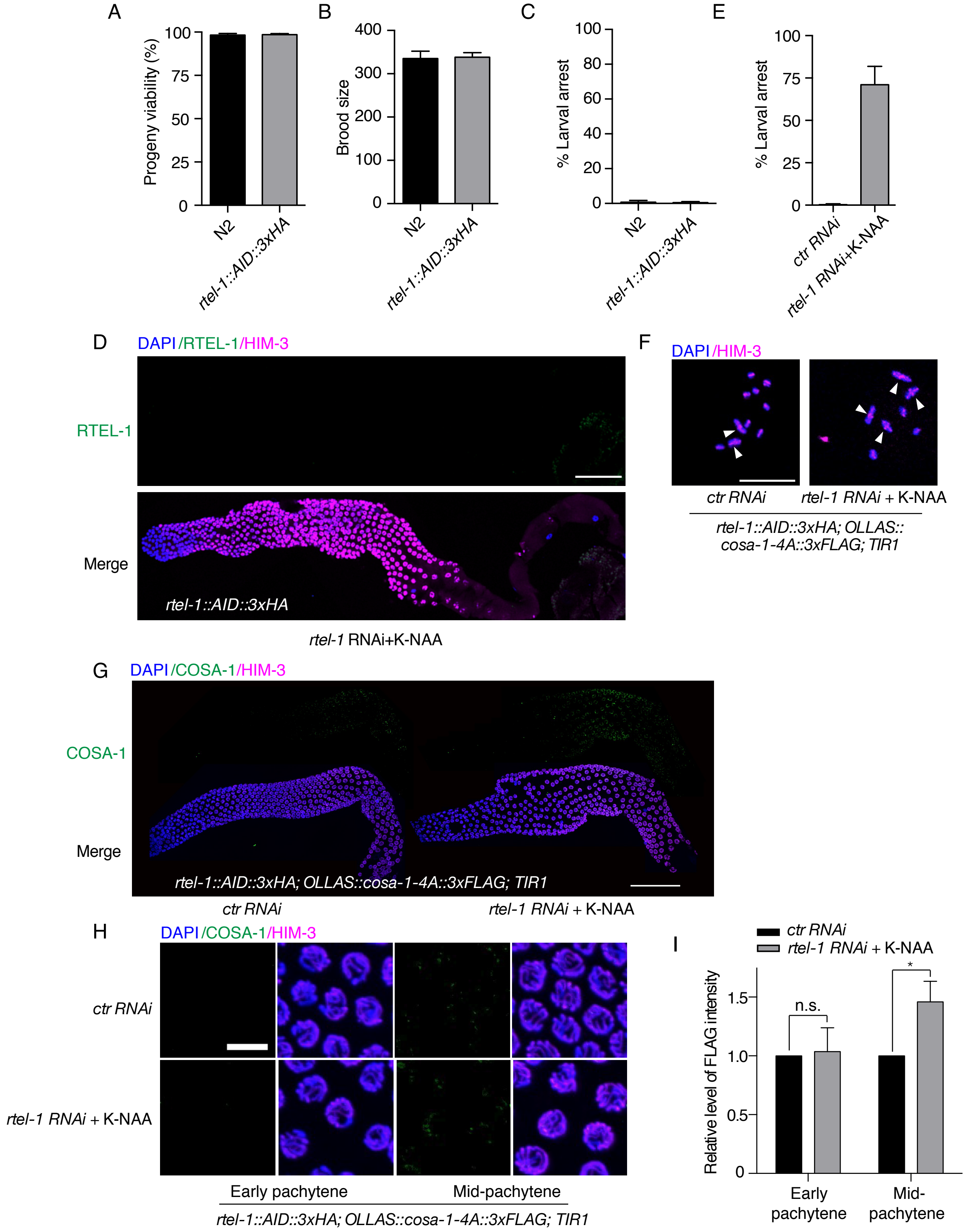

Figure S13 Analysis of rtel-1::AID::3xHA knock-in *C. elegans* strain

(A-C) Quantification of the progeny viability, brood size and offspring development of rtel-1::AID::3xHA knock-in C. elegans strain. (D) Whole gonad immunostaining with antibodies against HA to detect RTEL-1 (green) after depletion by RNAi and K-NAA-mediated degradation. Scalebar, 50 μm. (E) Depletion of RTEL-1 by RNAi and K-NAA-mediated degradation leads to larval arrest. (F) Depletion of RTEL-1 partially restored bivalents formation in cosa-1-4A mutants, as revealed by increased chiasmata (white arrowheads). scalebar 10 μm. (G) Whole gonad immunostaining with antibodies against FLAG to detect COSA-1 (green) after depletion of RTEL-1. Scalebar, 50 μm. (H) Representative images of early and mid-pachytene nuclei from control and RTEL-1 depleted germline stained with antibodies against FLAG to detect COSA-1 (green) and HIM-3 (magenta), counterstained with DAPI (blue). Scalebar, 5 μm. (I) Quantification of the FLAG signal (COSA-1) in nuclei from control and RTEL-1 depleted germline. Statistical analyses were conducted using T-test, * $p<0.05$.

Yang et al Figure S14

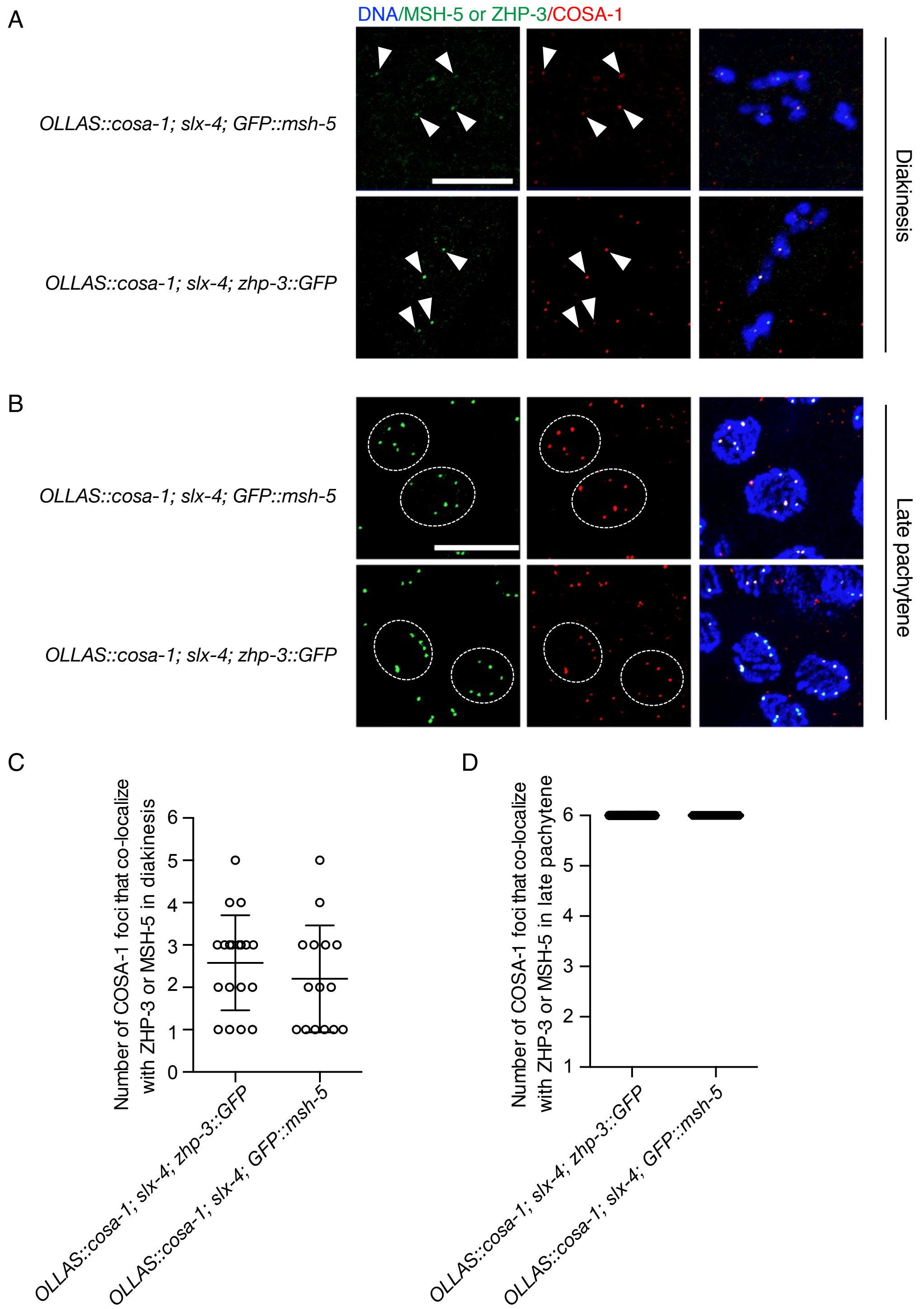

Figure S14

Analysis of MSH-5, ZHP-3 and COSA-1 foci in *slx-4* mutants

(A) Colocalization of COSA-1 with MSH-5 and ZHP-3 and foci at the junction of 'univalent pairs' in diakinesis oocytes of *slx-4* mutants. scalebar 10 μm. (B) MSH-5, ZHP-3 and COSA-1 foci are detected in late pachytene nuclei of *slx-4* mutants. scalebar 10 μm. (C) Quantification of COSA-1 foci colocalized with MSH-5 and ZHP-3 at the junction of 'univalent pairs' in diakinesis oocytes. (D) Quantification of COSA-1 foci colocalized with MSH-5 and ZHP-3 in late pachytene nuclei.

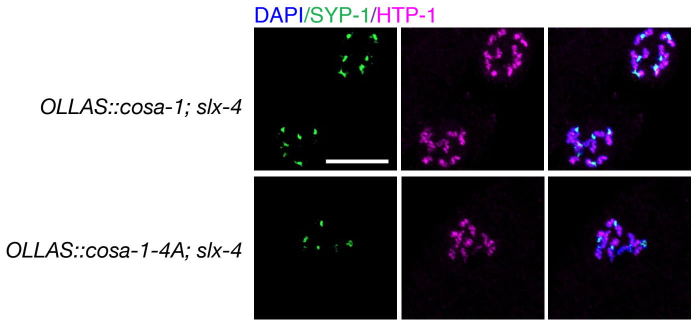

Figure S15

Chromosome remodeling is normal in *cosa-1-4A;slx-4* double mutants

scalebar 10 μm.

Supplementary Table 1

crRNAs, repair templates and genotyping primers for transgenes generated in this study

Allele	crRNAs and short/long repair templates & single-stranded templates	Genotyping primers and fragment sizes
OLLAS::cosa-1	5'- atcacgagaaacatggtgac-3'; 5'-aagtgtcaATGTCAAGTTCT -3';5'-TCCTGAGCCTCCctttcccatgaggcgtggtccgagttcgttagcgaatccgctCATtgacacttcagttcagttttcac -3'	F 5'-accaggtgtagtacggtagatt-3'; R 5'-acCTGATTCGCTGCTGATACG-3'; WT, 284 bp; Mutant, 326 bp
cosa-1-4A	5'- TCATATGACACGCTCCCCGA-3'; 5'-CAATGAGCCACCGAAAACGC -3';5'-ATCCGAATCTGAAGAAAAATGAGCCAAAAAGCGACAATGAGCCAgCagccACGCTGGTTTCAATGGccCCTGATgcTTATGACCCTCGGGGAGCGTGTCATATGATTTATTGGACGGATTGCATTGCACAAATGGCTGTTGATATTCGAG-3';5'-acagcaagaaaattgagagaaaactggctgaaaatggaaataattgattttttttgatttttaatttttcagACCGTAAAAAATCCGAATCTGAAGAAAAATGAGCCAAAAAGCGACAATGAGCCAgCagccACGCTGGTTTCAATGGccCCTGATgcTTATGACCCTCGGGGAGCGTGTCATATGATTTATTGGACGGATTGCATTGCACAAATGGCTGTTGATATTCGAGgtgtttatatcggaaattcaatgtttatattgcaaaaattcgtaaatttcaaggtttttaaccttaaaaattgggaaaattaggc -3'	F 5'-AGCGACAATGAGCCAgCagcc-3'; R 5'-CGGTGAATCGAACCTCATTGGG-3'; Mutant, 409 bp F 5'-AGCGACAATGAGCCACCGAAA-3'; R 5'-CGGTGAATCGAACCTCATTGGG-3'; WT, 409 bp
cosa-1-6A	5'- TCATATGACACGCTCCCCGA-3'; 5'-CAATGAGCCACCGAAAACGC -3';5'-ATCCGAATCTGAAGAAAAATGAGCCAAAAAGCGACAATGAGCCAgCagccACGCTGGTTTCAATGGcAgCcGcagcTTATGACCCTCGGGGAGCGTGTCATATGATTTATTGGACGGATTGCATTGCACAAATGGCTGTTGATATTCGAG -3'; 5'-acagcaagaaaattgagagaaaactggctgaaaatggaaataattgattttttttgatttttaatttttcagACC	F 5'-GTTTCAATGGcAgCcGcagc-3'; R 5'-CGGTGAATCGAACCTCATTGGG-3'; Mutant, 382 bp F 5'-GTTTCAATGGAACCTGATTT-3'; R 5'-

	GTAAAAAATCCGAATCTGAAGAAAAATGAGCCAAAAAGCGACAATGAGCCAgCagccACGCTGGTTTCAATGGcAgCcGcagcTTATGACCCTCGGGGAGCGTGTCATATGATTTATTGGACGGATTGCATTGCACAAATGGCTGTTGATATTCGAGgtgtttatatcggaaattcaatgtttatattgcaaaaattcgtaaatttcaaggtttttaaccttaaaaattgggaaaattaggc-3’	CGGTGAATCGAACCTCATTGGG-3’; WT, 382 bp
cosa-1$^{\Delta 41\text{-}53}$	5’- TCATATGACACGCTCCCCGA-3’; 5’-CAATGAGCCACCGAAAACGC-3’;5’-ATCCGAATCTGAAGAAAAATGAGCCAAAAAGCGACAATTATGACCCTCGGGGAGCGTGTCATATGATTTATTGGACGGATTGCATTGCACAAATGGCTGTTGATATTCGAG -3’; 5’-acagcaagaaaattgagagaaaactggctgaaaatggaaataattgatttttttttgattttttaatttttcagAACCGTAAAAAATCCGAATCTGAAGAAAAATGAGCCAAAAAGCGACAATTATGACCCTCGGGGAGCGTGTCATATGATTTATTGGACGGATTGCATTGCACAAATGGCTGTTGATATTCGAGgtgtttatatcggaaattcaatgtttatattgcaaaaattcgtaaatttcaaggttttttaaccttaaaaattgggaaaattaggc-3’	F 5’-GCAGCGAATCAGgttcgaaat-3’; R 5’-gaatttccgatataaacacCTCG-3’; WT, 304 bp; Mutant, 265 bp
cosa-1$^{\Delta 58\text{-}360}$	CRISPR/Cas9 screen identified a frameshift mutation in *cosa-1*, which had a stop codon at 58 site.	F 5’-GCAGCGAATCAGgttcgaaat-3’; R 5’-gaatttccgatataaacacCTCG-3’; WT, 304 bp; Mutant, 439 bp
cosa-1::3×FLAG	5’- cagagatggtagTTACGAGG-3’; 5’-GTATTCCGGAATGCAGCACCTCCTCGGGATCGGACTATAAAGATCACGACGGAGATTACAAGGACCATGATATCGACTACAAGGACGACGACGACAAGTAActaccatctctgacagcacctctttgtcgccgatt-3’;5’-GCGCGAAAAAGgtaactgctggccgagtttttctaggccacgcgtggcaattttacaattaattattttttatttatttcagAATGAGAGTATTCCGGAATGCAGCACCTCCTCGGGATCGGACTATAAAGATCACGACGGAGATTACAAGGACCATGATATCGACTACAAGGACGACGACGACAAGTAActaccatctctgacagca	F 5’-GTGACAATGCTTATGTCGAACCAT-3’; R 5’-gtggtgcaatgagtacgtgac-3’; WT, 407 bp; Mutant, 479 bp

	cctctttgtcgccgattccactggtcgcggctcgttcact<br>gcaacaaattattgatttttattgtcatgtaccatattgaa<br>tgcat -3’	
cosa-1::3×HA::Turbo ID	5’- cagagatggtagTTACGAGG-3’;<br>5’-TACCCATACGACGTCCCAGACTAC<br>GCCTACCCATATGATGTCCCGGATTA<br>CGCTTACCCATACGATGTTCCAGATTA<br>CGCTAAGGATAACACCGTCCCACTTA<br>AGCTTATCGCCCTTCTTGCTAACGGA<br>GAATTCCACTCTGGAGAGCAACTTG<br>GAGAGACTCTTGGAATGTCCCGTGC<br>TGCTATCAACAAGCATATCCAAACTCT<br>TCGTGATTGGGGAGTTGATGTTTTCA<br>CTGTTCCAGGTAAGTTTAAACATATAT<br>ATACTAACTAACCCTGATTATTTAAATT<br>TTCAGGAAAGGGATACTCTCTTCCAG<br>AGCCAATCCCACTTCTTAACGCTAAG<br>CAAATCCTTGGACAACTTGATGGAGG<br>ATCCGTCGCTGTCCTTCCAGTTGTTG<br>ATTCCACCAACCAATACCTTCTTGATC<br>GTATCGGAGAGCTTAAGTCTGGAGA<br>CGCTTGCATCGCTGAATACCAGCAG<br>GCTGGTAGAGGTAAGTTTAAACAGTT<br>CGGTACTAACTAACCATACATATTTAA<br>ATTTTCAGGAAGTCGTGGCCGTAAAT<br>GGTTTAGTCCTTTCGGCGCCAACCTC<br>TACCTTAGTATGTTTTGGCGTCTGAAA<br>AGAGGACCTGCGGCGATAGGCTTGG<br>GTCCAGTGATCGGTATTGTTATGGCC<br>GAGGCGCTGCGAAAGCTGGGAGCT<br>GATAAGGTTCGTGTTAAGTGGCCAAA<br>CGATCTTTACCTTCAAGACCGTAAGC<br>TTGCTGGAATCCTTGTCGAGCTTGCT<br>GGAATCACCGGAGACGCTGCTCAAA<br>TCGTTATCGGAGCTGGAATCAACGTT<br>GCTATGCGTCGTGTTGAGGTAAGTTT<br>AAACATGATTTTACTAACTAACTAATCT<br>GATTTAAATTTTCAGGAGTCTGTTGTT<br>AACCAAGGATGGATCACTCTTCAAGA<br>GGCTGGAATCAACCTTGATCGTAACA	F 5’-<br>GTGACAATGCTTA<br>TGTCGAACCAT-3’<br>; R 5’-<br>gtggtgcaatgagtacgt<br>gac-3’; WT, 407 bp;<br>Mutant, 1598 bp

	CCCTTGCTGCCACCCTTATCCGTGAG CTTCGTGCTGCCCTTGAACTTTTCGA GCAAGAGGGACTTGCTCCATACCTTC CACGTTGGGAGAAGCTTGATAACTTC ATCAACCGCCCAGTTAAGCTTATCAT CGGAGATAAGGAAATCTTCGGAATCT CTCGCGGAATCGATAAGCAAGGAGC TCTTCTTCTTGAGCAAGATGGAGTTA TTAAACCATGGATGGGAGGAGAAATT TCCCTTCGTTCCGCCGAGAAG -3’;5’-gcaaattttttgataaatcaatagtttcccggca attttttagcgggaattcaaatttcttcaaaaattttttgcg gaaattcaaattttttcagaaaagttttggcgggaaaat attggaatttcttaaaaaatttattggcggaaattcaaat ttcttcagaaaagttttggcgggaattcaaatttcttcac aaaaatttggagcaccttttctcaaattttgaaccaaa aattcacgaaattcgctattttggagcagttttttttgagt attttatatcaatttttcagATTAAACATCTCAAA ATTCAAATGGGATATCCAATTGCTCGC CGCCGCAACCGTCCAAACTGCTTAC ATTCTACTTCTCGGCACTTCTCAAATT GCCAATGTTTCAGTGATAATCAATAAT TTATTGAGATGTGACAATGCTTATGTC GAACCATTAAAGCAATCGATTATAGAG CTCGCCTGCGCGAAAAAGgtaactgctgg ccgagtttttctaggccacgcgtggcaattttacaatta attatttttatttatttcagAATGAGAGTATTCCG GAATGCAGCACCTCCTCGTACCCATA CGACGTCCCAGACTACGCCTACCCAT ATGATGTCCCGGATTACGCTTACCCA TACGATGTTCCAGATTACGCTAAGGAT AACACCGTCCCACTTAAGCTTATCGC CCTTCTTGCTAACGGAGAATTCCACT CTGGAGAGCAACTTGGAGAGACTCT TGGAATGTCCCGTGCTGCTATCAACA AGCATATCCAAACTCTTCGTGATTGG GGAGTTGATGTTTTCACTGTTCCAGG TAAGTTTAAACATATATATACTAACTAA CCCTGATTATTTAAATTTTCAGGAAAG GGATACTCTCTTCCAGAGCCAATCCC ACTTCTTAACGCTAAGCAAATCCTTG GACAACTTGATGGAGGATCCGTCGC TGTCCTTCCAGTTGTTGATTCCACCA ACCAATACCTTCTTGATCGTATCGGA	

	GAGCTTAAGTCTGGAGACGCTTGCAT<br>CGCTGAATACCAGCAGGCTGGTAGA<br>GGTAAGTTTAAACAGTTCGGTACTAA<br>CTAACCATACATATTTAAATTTTCAGG<br>AAGTCGTGGCCGTAAATGGTTTAGTC<br>CTTTCGGCGCCAACCTCTACCTTAGT<br>ATGTTTTGGCGTCTGAAAAGAGGACC<br>TGCGGCGATAGGCTTGGGTCCAGTG<br>ATCGGTATTGTTATGGCCGAGGCGCT<br>GCGAAAGCTGGGAGCTGATAAGGTT<br>CGTGTTAAGTGGCCAAACGATCTTTA<br>CCTTCAAGACCGTAAGCTTGCTGGAA<br>TCCTTGTCGAGCTTGCTGGAATCACC<br>GGAGACGCTGCTCAAATCGTTATCGG<br>AGCTGGAATCAACGTTGCTATGCGTC<br>GTGTTGAGGTAAGTTTAAACATGATTT<br>TACTAACTAACTAATCTGATTTAAATTT<br>TCAGGAGTCTGTTGTTAACCAAGGAT<br>GGATCACTCTTCAAGAGGCTGGAATC<br>AACCTTGATCGTAACACCCTTGCTGC<br>CACCCTTATCCGTGAGCTTCGTGCTG<br>CCCTTGAACTTTTCGAGCAAGAGGG<br>ACTTGCTCCATACCTTCCACGTTGGG<br>AGAAGCTTGATAACTTCATCAACCGC<br>CCAGTTAAGCTTATCATCGGAGATAA<br>GGAAATCTTCGGAATCTCTCGCGGAA<br>TCGATAAGCAAGGAGCTCTTCTTCTT<br>GAGCAAGATGGAGTTATTAAACCATG<br>GATGGGAGGAGAAATTTCCCTTCGTT<br>CCGCCGAGAAGTAActaccatctctgacagc<br>acctctttgtcgccgattccactggtcgcggctcgttca<br>ctgcaacaaattattgatttttattgtcatgtaccatattg<br>aatgcataatgtttaatttaataaatttggatttagttttat<br>aaatatgtttcatttttcaatgaaatcatccaaaaattca<br>aatttgcgcgtaaagtatggagttttcgcaatttcctgtc<br>acgtactcattgcaccacaatttacgcgcattatctcta<br>gaatttttgtagaaatcttaaattttgcgtgaaaaatagt<br>gattttttaacgaaaaattgtaaaaattcgaagaaagt<br>aaattttaaaaattaaatttgcgcgtaaagtatagtgtttt<br>aacatttttctgccacttatttaatacaccacatttcacgc<br>gcaaattcaggagtttt -3’	

cosa-1::3×FLAG::GFP nanobody	5’- cagagatggtagTTACGAGG-3’;<br>5’-GTATTCCGGAATGCAGCACCTCCTCGGGATCGGACTATAAAGATCACGACGGAGATTACAAGGACCATGATATCGACTACAAGGACGACGACGACAAGGGCTCAGATCAAGTCCAACTGGTGGAGTCTGGTGGCGCTTTGGTGCAGCCAGGTGGCTCTCTGCGTTTGTCCTGTGCCGCTTCTGGCTTCCCAGTGAACCGCTATTCCATGCGCTGGTATCGCCAGGCTCCAGGCAAAGAGCGTGAGTGGGTAGCCGGTATGTCCAGCGCGGGTGATCGTAGCTCCTATGAAGACTCCGTGAAGGGCCGTTTCACCATCAGCCGTGACGATGCCCGTAACACGGTGTATCTGCAAATGAACAGCTTGAAACCTGAAGATACGGCCGTGTATTACTGTAATGTGAACGTGGGCTTCGAGTATTGGGGCCAAGGCACCCAGGTCACCGTCTCCAGCTAActaccatctctgacagcacctctttgtc-3’;5’-GCGCGAAAAAGgtaactgctggccgagtttttctaggccacgcgtggcaattttacaattaattattttttatttatttcagAATGAGAGTATTCCGGAATGCAGCACCTCCTCGGGATCGGACTATAAAGATCACGACGGAGATTACAAGGACCATGATATCGACTACAAGGACGACGACGACAAGGGCTCAGATCAAGTCCAACTGGTGGAGTCTGGTGGCGCTTTGGTGCAGCCAGGTGGCTCTCTGCGTTTGTCCTGTGCCGCTTCTGGCTTCCCAGTGAACCGCTATTCCATGCGCTGGTATCGCCAGGCTCCAGGCAAAGAGCGTGAGTGGGTAGCCGGTATGTCCAGCGCGGGTGATCGTAGCTCCTATGAAGACTCCGTGAAGGGCCGTTTCACCATCAGCCGTGACGATGCCCGTAACACGGTGTATCTGCAAATGAACAGCTTGAAACCTGAAGATACGGCCGTGTATTACTGTAATGTGAACGTGGGCTTCGAGTATTGGGGCCAAGGCACCCAGGTCACCGTCTCCAGCTAActaccatctctgacagcacctctttgtcgccgattccactggtcgcggctcgttcactgcaacaaattattgatttttattgtcatgtaccatattgaatgcat -3’	F 5’-GTGACAATGCTTATGTCGAACCAT-3’; R 5’-gtggtgcaatgagtacgtgac-3’; WT, 407 bp; Mutant, 833 bp

msh-5::AID::3×HA	5’-CGAACGATCTATCGTCTCAT-3’;<br>5’-GCaGATGAaACtATAGATCGTTCGAAAAGAAGTGGAGGCTCAGGAatgcctaaagatccagccaaacctccggccaaggcacaagttgtgggatggccaccggtgagatcataccggaagaacgtgatggtttcctgccaaaaatcaagcggtggcccggaggcggcggcgttcgtgaagGGAGGCTCAGGATACCCATACGACGTCCCAGACTACGCCTACCCATATGATGTCCCGGATTACGCTTACCCATACGATGTTCCAGATTACGCTTAA<br>-3’;5’-GAGGAAGATGATGAGTTCTTGAAGAGTTTCCTTGAAACAGAAGGATCTCTCCATATCGATACGAGCGCaGATGAaACtATAGATCGTTCGAAAAGAAGTGGAGGCTCAGGAatgcctaaagatccagccaaacctccggccaaggcacaagttgtgggatggccaccggtgagatcataccggaagaacgtgatggtttcctgccaaaaatcaagcggtggcccggaggcggcggcgttcgtgaagGGAGGCTCAGGATACCCATACGACGTCCCAGACTACGCCTACCCATATGATGTCCCGGATTACGCTTACCCATACGATGTTCCAGATTACGCTTAAttttatataattagaatttcgtatttcttgtaatgttcaatcttgttcaatgtatttcgttttcgtttttaaattatttatagcgcattttaataaatac -3’	F 5’-GAAAAGAATTGCAGTGCTCCTGT-3’; R 5’-cgttgtgaactattgcacgag-3’; WT, 286 bp; Mutant, 526 bp
rtel-1::AID::3×HA	5’- TTGCTCCAACGTTCTCCGAT-3’;<br>5’-ttaattttccagCTTGCTCCAACGTTCTCCGATCaGAAGACAAAATGAAATATCTGAAAAAGGCGCTTGAATCGAAGATCCACACAGGAGGCTCAGGAatgcctaaagatccagccaaacctccggccaaggcacaagttgtgggatggccaccggtgagatcataccggaagaacgtgatggtttcctgccaaaaatcaagcggtggcccggaggcggcggcgttcgtgaagGGAGGCTCAGGATACCCATACGACGTCCCAGACTACGCCTACCCATATGATGTCCCGGATTACGCTTACCCATACGATGTTCCAGATTACGCTTAA<br>-3’;5’-TGTTCCGCACAAGGCTGATTTGTTTATTGgtttgttagaattcaaatttttatttcaacaaaaaaatacaattaattttccagCTTGCTCCAACGTTCTCCGATCaGAAGACAAAATGAAATATCTGAAAAAGGCGCTTGAATCGA	F 5’-GCTGAAAGTATCAGATGGGATG-3’; R 5’-tgggaagtgaccatgagatcgcat-3’; WT, 342 bp; Mutant, 582 bp

	AGATCCACACAGGAGGCTCAGGAatg cctaaagatccagccaaacctccggccaaggcaca agttgtgggatggccaccggtgagatcataccggaa gaacgtgatggtttcctgccaaaaatcaagcggtggc ccggaggcggcggcgttcgtgaagGGAGGCT CAGGATACCCATACGACGTCCCAGAC TACGCCTACCCATATGATGTCCCGGA TTACGCTTACCCATACGATGTTCCAGA TTACGCTTAAttttcatttattttttttctctcgtaacat ttcctaactctctagatccttttacatgtaaaacattattg accggtgtttaat -3’	
msh-5-T1009A	5’-TCACCCATTTGGATTGGAGT-3’; single-stranded templates:5’-ACCACCAGCTTCACCCATTTGGATaG GtGccGGAATATGTATTGCAGTTCGAG TCGAG -3’;	F 5’-attaggtctcaaatcgag agg -3’; R 5’-TCACCCATTTGGA TaGGtccc -3’; Mutant, 239 bp F 5’-attaggtctcaaatcgag agg -3’; R 5’-TCACCCATTTGGA TTGGAcTT -3’; WT, 239 bp
3×FLAG::dsb-2	5’-agaaaaaaaATGAGTGCACG -3’; 5’-GGATCGGACTATAAAGATCACGAC GGAGATTACAAGGACCATGATATCGA CTACAAGGACGACGACGACAAGGGA GGCTCAGGATCCACT -3’; 5’-gtttatttcaataaatatctcaatttttacagaaaaaa aATGGGATCGGACTATAAAGATCACG ACGGAGATTACAAGGACCATGATATC GACTACAAGGACGACGACGACAAGG GAGGCTCAGGATCCACTAGTGCACG TGGCCTGAAAGTTGAGATGTACTACA AATTAGC -3’;	5’-aatggctgttaccgtagtt gt-3’; 5’-acgcgaaaattacC TCTGCTTC -3’; WT, 186 bp; Mutant,276 bp
cdk-2::AID::3×FLAG	5’-acacgatgtttaggttatgg -3’; 5’-GGAGGCTCAGGAatgcctaaagatccagcc aaacctccggccaaggcacaagttgtgggatggcc accggtgagatcataccggaagaacgtgatggtttcc tgccaaaaatcaagcggtggcccggaggcggcgg cgttcgtgaagGGATCGGACTATAAAGATC ACGACGGAGATTACAAGGACCATGAT ATCGACTACAAGGACGACGACGACA	5’-GCGCAGAACA GGgtaaag -3’; 5’-gaggaaagactcggta aaaagag-3’; WT, 176 bp; Mutant, 398bp

	AGGGA-3’;<br>5’-CTGCTGAACAATCATCAGGAGAAGTCAATCTTCGGAGGCTCAGGAatgcctaaagatccagccaaacctccggccaaggcacaagttgtgggatggccaccggtgagatcataccggaagaacgtgatggtttcctgccaaaaatcaagcggtggcccggaggcggcggcgttcgtgaagGGATCGGACTATAAAGATCACGACGGAGATTACAAGGACCATGATATCGACTACAAGGACGACGACGACAAGGGATAAtatatcatgtcctccataacctaaacatcgtgtactat -3’;	
zhp-3::AID::3× HA	5’-gagattaaaacaTTAATCGG -3’;<br>5’-GGAGGCTCAGGAatgcctaaagatccagccaaacctccggccaaggcacaagttgtgggatggccaccggtgagatcataccggaagaacgtgatggtttcctgccaaaaatcaagcggtggcccggaggcggcggcgttcgtgaagGGAGGCTCAGGATACCCATACGACGTCCCAGACTACGCCTACCCATATGATGTCCCGGATTACGCTTACCCATACGATGTTCCAGATTACGCTTAA -3’;<br>5’-GGAAACCGATCAATGGTCGGAGCTTCATTGGACCCGCCGATGGAGGCTCAGGAatgcctaaagatccagccaaacctccggccaaggcacaagttgtgggatggccaccggtgagatcataccggaagaacgtgatggtttcctgccaaaaatcaagcggtggcccggaggcggcggcgttcgtgaagGGAGGCTCAGGATACCCATACGACGTCCCAGACTACGCCTACCCATATGATGTCCCGGATTACGCTTACCCATACGATGTTCCAGATTACGCTTAAtgttttaatctcgtttttttctgaattcgttctttatttg-3’;	5’-cgttttcagGATACCTCGCAC -3’;<br>5’-tcagatgtgaactaggtagag -3’;<br>WT, 207 bp;<br>Mutant, 447 bp

Supplementary Table 2

List of strains

Strain #	Strains	Source
sYH_0007	N2	Caenorhabditis Genetics Center
sYH_0067	*meIs8[pie-1p::GFP::cosa-1, unc-119(+)] II*	Yokoo et al., 2012
sYH_0087	*zhp-3::GFP V*	Bhalla et al, 2018
sYH_0224	*OLLAS::cosa-1 III*	This study, using the strategy of Janisiw et al, 2018
sYH_0229	*cdk-2::AID::3xFLAG I*	This study, using the Haversat et al, 2022
sYH_0059	*GFP::msh-5 IV*	Janisiw et al, 2018
sYH_0008	*GFP::him-6 IV*	Caenorhabditis Genetics Center
sYH_0293	*OLLAS::cosa-1-4A/qC1 III The P43K44E50F53 of cosa-1 mutated into alanine.*	This study
sYH_0294	*OLLAS::cosa-1-6A/qC1 III The P43K44E50P51D52F53 of cosa-1 mutated into alanine.*	This study
sYH_0292	*OLLAS::cosa-1*$^{\Delta 41\text{-}53}$ */qC1 III Truncated mutation of cosa-1 at 41-53 AA*	This study
sYH_0305	*OLLAS::cosa-1*$^{\Delta 58\text{-}360}$ */qC1 III Frameshift mutation in cosa-1, which had a stop codon at 58 site.*	This study
sYH_0289	*MEIS8[PIE-1P::GFP::cosa-1, UNC-119(+)] II;OLLAS::cosa-1-4A III*	This study
sYH_0290	*MEIS8[PIE-1P::GFP::cosa-1, UNC-119(+)] II;OLLAS::cosa-1-6A III*	This study
sYH_0291	*MEIS8[PIE-1P::GFP::cosa-1, UNC-119(+)] II; OLLAS::cosa-1*$^{\Delta 41\text{-}53}$ *III*	This study
sYH_0308	*MEIS8[PIE-1P::GFP::cosa-1, UNC-119(+)] II; OLLAS::cosa-1*$^{\Delta 58\text{-}360}$ *III*	This study
sYH_0240	*OLLAS::cosa-1 III; GFP::msh-5 IV*	This study
sYH_0297	*OLLAS::cosa-1-4A/qC1 III; GFP::msh-5 IV*	This study
sYH_0298	*OLLAS::cosa-1-6A/qC1 III; GFP::msh-5 IV*	This study
sYH_0329	*OLLAS::cosa-1*$^{\Delta 58\text{-}360}$*/qC1 III; GFP::msh-5 IV*	This study
sYH_0241	*OLLAS::cosa-1III; zhp-3::GFP V*	This study
sYH_0299	*OLLAS::cosa-1-4A/qC1 III; zhp-3::GFP V*	This study
sYH_0300	*OLLAS-cosa-1-6A/qC1 III; zhp-3-GFP V*	This study

sYH_0330	*OLLAS::cosa-1*$^{\Delta 58\text{-}360}$*/qC1 III; zhp-3::GFP V*	This study
sYH_0402	*cdk-2::AID::3xFLAG I; OLLAS::cosa-1 III*	This study
sYH_0403	*cdk-2::AID::3xFLAG I; OLLAS::cosa-1-4A/qC1 III*	This study
sYH_0404	*cdk-2::AID::3xFLAG I; OLLAS::cosa-1-6A/qC1 III*	This study
sYH_0415	*cdk-2::AID::3xFLAG I; OLLAS::cosa-1*$^{\Delta 58\text{-}360}$*/qC1 III*	This study
sYH_0432	*cosa-1::3×HA::TurboID III*	This study
sYH_0433	*cdk-2::AID::3xFLAG I; cosa-1::3×HA::TurboID III*	This study
sYH_0607	*cosa-1::3×HA::TurboID III; GFP::him-6 IV*	This study
sYH_0433	*cdk-2::AID::3xFLAG I; cosa-1::3×HA::TurboID III*	This study
sYH_0389	*OLLAS::cosa-1::3×FLAG III*	This study
sYH_0416	*OLLAS::cosa-1-4A::3×FLAG/qC1 III*	This study
sYH_0417	*OLLAS::cosa-1-6A::3×FLAG/qC1 III*	This study
sYH_0526	*OLLAS::cosa-1-4A::3×FLAG::GFP nanobody/qC1 III*	This study
sYH_0534	*OLLAS::cosa-1-4A::3×FLAG::GFP nanobody/qC1 III ; GFP::msh-5/nt1 IV*	This study
sYH_0553	*OLLAS::cosa-1-4A::3×FLAG::GFP nanobody/qC1 III ; zhp-3::GFP V*	This study
sYH_0569	*OLLAS::cosa-1::3×FLAG::GFP nanobody III*	This study
sYH_0572	*OLLAS::cosa-1::3×FLAG::GFP nanobody III; zhp-3::GFP V*	This study
sYH_0573	*OLLAS::cosa-1::3×FLAG::GFP nanobody III; GFP::msh-5/nt1 IV*	This study
sYH_0527	*rtel-1::AID::3×HA I*	This study
sYH_0532	*rtel-1::AID::3×HA I; ieSi38 [sun-1p::TIR1::mRuby::sun-1 3'UTR + Cbr-unc-119(+)] IV*	This study
sYH_0533	*rtel-1::AID::3×HA I; OLLAS::cosa-1-4A::3×FLAG/qC1 III; ieSi38 [sun-1p::TIR1::mRuby::sun-1 3'UTR + Cbr-unc-119(+)]IV*	This study
sYH_0512	*msh-5::AID::3×HA IV*	This study
sYH_0525	*msh-5::AID::3×HA IV; ieSi38 [sun-1p::TIR1::mRuby::sun-1 3'UTR + Cbr-unc-119(+)] IV*	This study
sYH_0070	*him-18(tm2181)/qC1 III; GFP::him-6 IV*	This study
sYH_0071	*MeIs8[pie-1p::GFP::cosa-1, unc-119(+)] II; him-18(tm2181)/qC1 III*	This study
sYH_0072	*him-18(tm2181)/qC1 III; zhp-3::GFP V*	This study
sYH_0073	*him-18(tm2181)/qC1 III; GFP::msh-5 IV*	This study
sYH_0242	*OLLAS::cosa-1 III; him-18(tm2181) III /qC1; GFP::msh-5 IV*	This study
sYH_0243	*OLLAS::cosa-1 III; him-18(tm2181) III /qC1; zhp-3::GFP V*	This study
sYH_0324	*OLLAS::cosa-1-4A III; him-18(tm2181) III /qC1*	This study
sYH_0253	*OLLAS::cosa-1 III; him-18(tm2181) III /qC1*	This study

sYH_0177	*mus-81 (tm1937) /ht2 I; xpf-1(TG1660) II; zhp-3::GFP V*	This study
sYH_0633	*zhp-3::AID::3×HA I*	This study
sYH_0655	*zhp-3::AID::3×HA I; OLLAS::cosa-1::3×FLAG III*	This study
sYH_0656	*zhp-3::AID::3×HA I; OLLAS::cosa-1-4A::3×FLAG /qC1III*	This study
sYH_0657	*zhp-3::AID::3×HA I; OLLAS::cosa-1-6A::3×FLAG /qC1III*	This study
sYH_0658	*zhp-3::AID::3×HA I; OLLAS::cosa-1*$^{\Delta 58\text{-}360}$ */qC1 III*	This study
sYH_0648	*3×FLAG::dsb-2 I*	This study
sYH_0649	*3×FLAG::dsb-2 I; OLLAS::cosa-1 III*	This study
sYH_0650	*3×FLAG::dsb-2 I; OLLAS::cosa-1-4A /qC1III*	This study
sYH_0651	*3×FLAG::dsb-2 I; OLLAS::cosa-1-6A /qC1III*	This study
sYH_0652	*3×FLAG::dsb-2 I; OLLAS::cosa-1*$^{\Delta 58\text{-}360}$ */qC1III*	This study
sYH_0678	*msh-5-T1009A::AID::3×HA IV*	This study
sYH_0679	*OLLAS::cosa-1::3×FLAG III; msh-5-T1009A::AID::3×HA IV*	This study
sYH_0680	*OLLAS::cosa-1-4A::3×FLAG/qC1 III; msh-5-T1009A::AID::3×HA IV*	This study
sYH_0681	*OLLAS::cosa-1::3×FLAG III;GFP::him-6 IV; msh-5::AID::3×HA IV*	This study
sYH_0682	*OLLAS::cosa-1-4A::3×FLAG/qC1 III; GFP::him-6 IV; msh-5::AID::3×HA IV*	This study
sYH_0100	*mls12* (CB5584)	Caenorhabditis Genetics Center
sYH_0124	Hawaii (CB4856)	Caenorhabditis Genetics Center
sYH_0701	*zhp-3::GFP V* (Hawaii)	This study
sYH_0702	*OLLAS::cosa-1-4A::3×FLAG::GFP nanobody/qC1III* (Hawaii)	This study
sYH_0703	*OLLAS::cosa-1-4A::3×FLAG::GFP nanobody/ qC1III; zhp-3::GFP V* (Hawaii)	This study
sYH_0524	*OLLAS::cosa-1::3×FLAG III; msh-5::AID::3×HA IV*	This study
sYH_0666	*OLLAS::cosa-1-4A::3×FLAG/qC1III; msh-5::AID::3×HA IV*	This study
sYH_0667	*OLLAS::cosa-1-6A::3×FLAG/qC1III; msh-5::AID::3×HA IV*	This study
sYH_0668	*OLLAS::cosa-1*$^{\Delta 58\text{-}360}$*/qC1III; msh-5::AID::3×HA IV*	This study
